# Characterizing Eckol as a Therapeutic Aid: A Systematic Review

**DOI:** 10.3390/md17060361

**Published:** 2019-06-18

**Authors:** Bandana Manandhar, Pradeep Paudel, Su Hui Seong, Hyun Ah Jung, Jae Sue Choi

**Affiliations:** 1 Department of Food and Life Science, Pukyong National University, Busan 48513, Korea; manandhar269@gmail.com (B.M.); phr.paudel@gmail.com (P.P.); seongsuhui@naver.com (S.H.S.); 2 Department of Food Science and Human Nutrition, Chonbuk National University, Jeonju 54896, Korea

**Keywords:** seaweed, eckol, phlorotannin, *Ecklonia*, bioactivity

## Abstract

The marine biosphere is a treasure trove of natural bioactive secondary metabolites and the richest source of structurally diverse and unique compounds, such as phlorotannins and halo-compounds, with high therapeutic potential. Eckol is a precursor compound representing the dibenzo-1,4-dioxin class of phlorotannins abundant in the *Ecklonia* species, which are marine brown algae having a ubiquitous distribution. In search of compounds having biological activity from macro algae during the past three decades, this particular compound has attracted massive attention for its multiple therapeutic properties and health benefits. Although several varieties of marine algae, seaweed, and phlorotannins have already been well scrutinized, eckol deserves a place of its own because of the therapeutic properties it possesses. The relevant information about this particular compound has not yet been collected in one place; therefore, this review focuses on its biological applications, including its potential health benefits and possible applications to restrain diseases leading to good health. The facts compiled in this review could contribute to novel insights into the functions of eckol and potentially enable its use in different uninvestigated fields.

## 1. Introduction

Seaweed refers to a diverse group of macroscopic, multicellular, and marine algae. Marine algae have been consumed as sea vegetables for the diet and ingredients in medicine since ancient times in countries including Korea, China, and Japan [1]. One of the earth’s affluent sources of natural antioxidants and antimicrobials are seaweeds [2,3,4,5]. They also contain a rich variety of vitamins, such as A, B_l_, B_12_, C, D, and E; riboflavin; niacin; pantothenic acid; and folic acid, as well as minerals, such as calcium, phosphorus, sodium, potassium, and iodine [6]. Polysaccharides (laminarins and fucoidans), polyphenols such as phlorotannins [7], carotenoid pigments such as fucoxanthin [8] and astaxanthin, sterols, and mycosporine-like amino acids have been isolated from seaweeds in the past.

Among the marine seaweeds, brown algae, including *Ecklonia* species and *Ishige okamurae*, have been considerably evaluated as these species contains phlorotannins [9]. Phlorotannins with both phenyl and phenoxy units having different molecular weights are present in the marine brown algae [10]. According to their linkages, phlorotannins can be characterized into fuhalols and phlorethols having an ether linkage, fucols having a phenyl linkage, fucophloroethols with an ether and phenyl linkage, and eckols having a dibenzodioxin linkage. *Ecklonia* is the most abundant genus of kelp (brown algae) associated with the Lessoniaceae family having an affluence of eckol-type phlorotannins.

**Figure 1:**
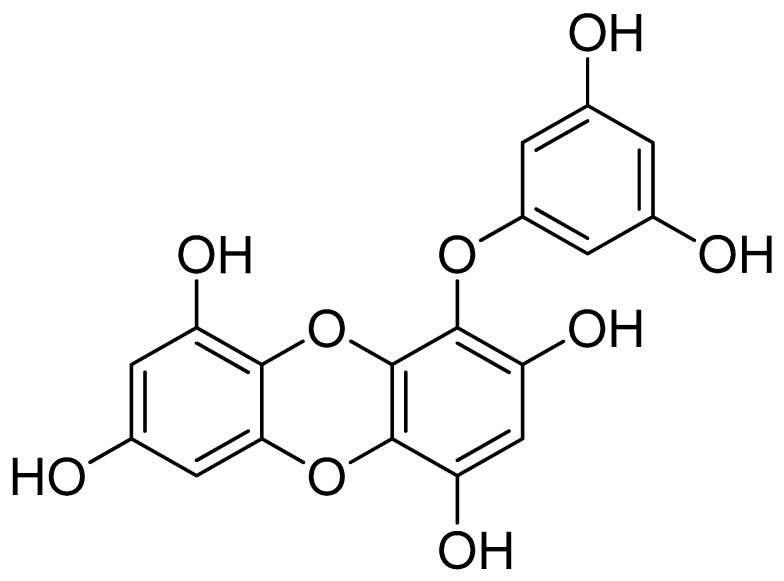
Structure of eckol.

**Figure 2:**
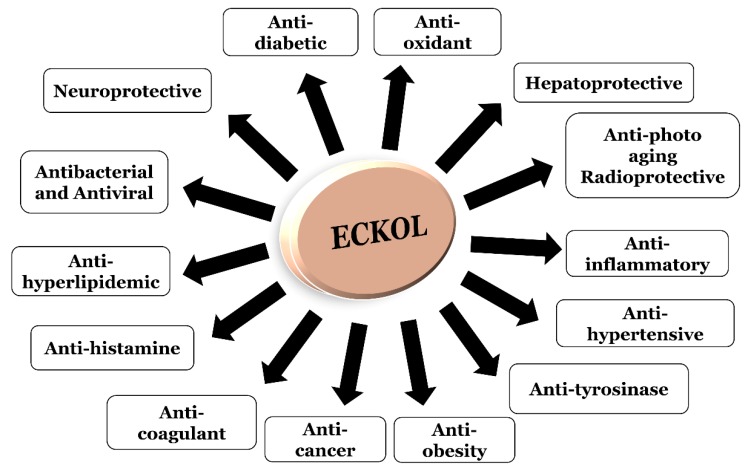
Visual representation of the biological activities of eckol.

Eckol, a precursor compound illustrating the dibenzo-1,4-dioxin class of phlorotannins, contains phloroglucinol components linked to each other in multiple fashions (Figure 1). Several marine organisms are known to produce eckol, particularly in brown (Table 1) and red algae [10]. Eckol has been shown to exhibit antioxidant [13,21,22], anti-inflammatory [23,24], hepatoprotective [14,25], neuroprotective [26], anti-obesity [27], anti-hypertensive [28], and antibacterial and antiviral [29] activity. Because of these numerous health benefits, this particular compound has been a prime focus for researcher eager to elucidate its pharmacological potential (Table 2 and Figure 2).

Therefore, understanding the biological activities of eckol to expand effective therapeutic approaches is very important. Although abundant information related to compounds from marine algae [5,30,31,32], seaweed [6,33,34,35,36,37,38], and phlorotannins from seaweeds [10,39,40] is accessible, little information is available about this particular type of phlorotannin. Therefore, this review summarizes the biological characterization and pharmacological bioactivity of eckol, focusing on recent advancements related to this particular compound. 

## 2. Biological Activities of Eckol

### 2.1. Anti-Oxidant Activity

The lack of balance between antioxidants and free-radicals in the body leads to oxidative stress. As free radicals react easily with other molecules in the human body, they can lead to large-chain chemical reactions called oxidation which can be useful or harmful.

Human cells remove the ROS through their defense system by the mode of enzymatic and non-enzymatic pathways. When a body contains free reactive oxygen species (ROS) beyond its defensive capacity, the ROS can start damaging fatty tissue, nucleic acids, and proteins, which can give rise to a vast sum of diseases over time, including neurological disorders [83,84], diabetes [85], cancer [86], and dermal diseases [87].

Manganese superoxide dismutase (MnSOD) is an antioxidant mitochondrial enzyme in exhibiting cytoprotective effects against oxidative stress. This enzyme is used as a marker to evaluate the antioxidant properties of phytochemicals. As an antioxidant, eckol at 10 µg/mL recovered MnSOD expression and activity that had been decreased by 600 µM hydrogen peroxide (H_2_O_2_). Through the pathway of phosphorylated AMP-activated protein kinase/forkhead box O3a (AMPK/FoxO3a), the MnSOD expression was induced by eckol. Eckol-stimulated MnSOD expression was reduced by 10–50 nM of specific silencing RNAs (siRNAs) against FoxO3a and AMPK. Additionally, the cytoprotective activity of eckol against H_2_O_2_-provoked cell death was reduced by diethyldithiocarbamate (a MnSOD inhibitor). Thus, by activating the AMPK/FoxO3a-mediated induction of MnSOD, eckol protected the cells against the oxidative stress in mitochondria [44].

Similarly, there was an induction of heme oxygenase (HO)-1 mRNA and protein expression in the Chinese hamster lung fibroblast (V79-4) cells in a concentration- and time-dependent manner, due to eckol, which resulted in high HO-1 activity. Enhancement of the phosphorylated form, nuclear translocation, antioxidant response element (ARE)-binding, and transcriptional activity of Nrf2 was observed in V79-4 cells upon exposure to 10 µg/mL of eckol. Furthermore, Nrf2 mediated the eckol-induced activation of the HO-1 promoter. The Nrf2 activation and induction of HO-1 expression were due to the contribution of the extracellular related kinase (Erk) and phosphoinositide 3-kinase/protein kinase B (PI3K/PKB). To further assess the likeliness of the cytoprotective activity of eckol due to HO-1 induction, the V79-4 cells were pretreated with HO-1 inhibitor zinc protoporphyrin (10 µM) and transfected with HO-1 siRNA (10–50 nM) and Nrf2 siRNA (10–50 nM). All of them markedly reduced the cytoprotective effect of eckol against H_2_O_2_-induced cell damage suggesting the involvement of Nrf2 transcription factor in eckol-mediated HO-1 induction. Additionally, the cytoprotective effect of eckol was reduced by U0126 (an inhibitor of ERK kinase) (10 nM) and LY294002 (an inhibitor of PI3K) (5 μM). Therefore, eckol has the ability to attenuate oxidative stress-induced cell death through Nrf2-mediated HO-1 activation via Erk and PI3K/Akt signaling [42].

Eckol isolated from *Ecklonia cava* had a cytoprotective effect against oxidative stress-induced cell damage in V79-4 cells. Eckol scavenged 2,2-diphenyl-1-picrylhydrazyl (DPPH) radicals, hydrogen peroxide (H_2_O_2_), hydroxy radicals, and intracellular ROS, with dose-dependent quenching effects. At 30 μM, eckol demonstrated a 79% radical scavenging effect on intracellular ROS. Eckol also prevented lipid peroxidation (31% at 30 μM) in a thiobarbituric acid reactive substances (TBARS) assay, reducing cell death in V79-4 cells which was induced by H_2_O_2_. After eckol treatment, there was a decrease in nuclear fragmentation and the apoptotic sub-G_1_ DNA content in V79-4 cells induced by H_2_O_2_. In addition, 30 μM eckol exhibited 47% and 43% ROS scavenging activity in cells damaged via serum starvation and γ-radiation, respectively. Eckol also influenced the activity of catalase by increasing it in a dose-dependent manner through the activation of phosphorylated ERK and NF-κB. Furthermore, eckol also increased the transcriptional activity of NF-κB. Hence, eckol is a capable of exhibiting cellular anti-oxidant activity and cytoprotection [43].

The antioxidant activity of five compounds, phloroglucinol, eckol, phlorofucofuroeckol A, dieckol, and 8,8´-bieckol from *Eisenia bicyclis* was examined by the weight gain test [18]. At a concentration of 0.05%, a mixture of eckol and phlorofucofuroeckol A was as effective as α-tocopherol. Although phloroglucinol showed low activity at 0.05%, it was most effective at 0.5%. The antioxidant activity of the phlorotannins seemed to depend on the degree of polymerization of the phloroglucinol, suggesting that phlorotannins of lower molecular weights (eckol and phlorofucofuroeckol A) were more potent as compared to higher weights (dieckol and 8,8´-bieckol).

A recent study evaluated *E. cava* extract on its antioxidant potential in airborne particulate matter (PM) (diameter < 10 μm (PM_10_)) exposed cultured keratinocytes. The cultured HaCaT keratinocytes exposed to PM_10_ in the presence and absence of *E. cava* extract were evaluated for their cell viability and cellular lipid peroxidation. Human epidermal keratinocytes exposed to PM_10_ (100 µg/mL) were used to examine the action of eckol and dieckol on cellular lipid peroxidation and cytokine expression.. There was a decrease in cell viability and an increase in lipid peroxidation when HaCaT cells were exposed to PM_10_, which was attenuated by *E. cava* extract (25, 50, 75 or 100 µg/mL) and its ethyl acetate (EtOAc) fraction (100 µg/mL). As compared to eckol (3 µg/mL), dieckol (10 µg/mL) attenuated cellular lipid peroxidation more effectively in both HaCaT cells and human epidermal keratinocytes. The inflammatory cytokines (TNF-α, IL-1β, IL-6, and IL-8) expression was also attenuated by dieckol and eckol in human epidermal keratinocytes stimulated with PM_10_ [12].

### 2.2. Anti-Diabetic Activity

Diabetes mellitus describes a group of diseases that affect how bodies use blood sugar (glucose). Chronic diabetic conditions include type 1 diabetes (T1DM) and type 2 diabetes (T2DM). With T1DM, the body does not make insulin, and in the more common T2DM (insulin-resistant state), the body fails to produce enough insulin or fails to respond adequately to insulin, leading to high glucose concentrations in the blood. The occurrence of severe hypoglycemia is related to intensive insulin therapy leading to research into antihyperglycemic agents focusing on traditional medicinal plants, as they could provide superior treatment as compared to synthetic drugs [88]. The methanol extract of *Ecklonia maxima*, four solvent-soluble fractions and three phlorotannins, phloroglucinol, dibenzo-1,4-dioxine-2,4,7,9-tetraol, and eckol were checked for their alpha-glucosidase inhibitory activity. The phloroglucinol derivative dibenzo-1,4-dioxine-2,4,7,9-tetraol (IC_50_ 33.693 ± 0.61 µM) and eckol (IC_50_ 11.163 ± 0.29 µM) displayed better inhibition than the positive control, acarbose (IC_50_ 1013.35 ± 89.01 µM), showing eckol to be a potential compound for the treatment of hyperglycemia [17]. 

Similarly, the *Ecklonia stolonifera-*derived EtOAc fraction exhibited inhibitory effects against advanced glycation end-products (AGEs) formation and rat lens aldose reductase (RLAR) and yielded phloroglucinol, dioxinodehydroeckol, eckol, phlorofucofuroeckol-A, dieckol, triphlorethol-B, 2-phloroeckol, and 7-phloroeckol [46]. The *E. stolonifera*-derived phlorotannins exhibited RLAR inhibitory activity, with eckol showing an IC_50_ of 54.68 µM, although most of the isolated phlorotannins showed no activity in the AGEs inhibition assay. These results were in contrast to another study [89] in which the inhibitory effect of the isolated compounds on glycation, was 96.2% for eckol, 86.7% for dieckol, and 76.0% for aminoguanidine at 1 mM. According to Jung et al. [46], the reasons for the discrepant results could include differences in the glycation conditions and glycation steps and the detectors used in the fluorescence, ELISA, and Western blot analyses. The α-amylase inhibition of the compounds was also checked where the percent inhibitions were found to be 87.5% for eckol at 1 mM. So, according to the results, eckol could be a promising treatment agent for diabetic complications and may possess significant inhibitory effects against AGEs, RLAR, and α-amylase [89].

Further insight into the anti-diabetic potential of eckol was provided by another study in which the methanolic extracts of *E. stolonifera* and *E. bicyclis*, fractions, and six isolated phlorotannins were tested against α-glucosidase and protein tyrosine phosphatase (PTP1B) enzymes. Eckol was characterized as a potent PTP1B inhibitor (IC_50_ 2.64 µM) and a moderately potent α-glucosidase inhibitor (IC_50_ 22.78 µM) [90]. Hence, these results suggest eckol as a potential therapeutic agent for controlling postprandial blood glucose levels, thereby preventing diabetic complications.

### 2.3. Hepatoprotective Activity

As the liver operates by making proteins and blood clotting factors, producing triglycerides, cholesterol, bile, and synthesizing glycogen, it is one of the most important organs. The ability to prevent hepatic damage is vitally important as it is involved in the detoxification of xenobiotics and drugs [91].

An acute liver injury model in mice induced by carbon tetrachloride (CCl_4_) was used to investigate the protective effects of eckol in addition to the possible mechanisms. The CCl_4_-induced rise in the serum levels of alanine transaminase (ALT) and (49.43 ± 16.03 U/L) and aspartate aminotransferase (AST) (63.21 ± 18.89 U/L) were suppressed by the pretreatment of eckol at doses of 0.5 and 1.0 mg/kg/day for 7 days with the amelioration of morphological liver injury. Terminal deoxynucleotidyl transferase-mediated dUTP nick end labelling (TUNEL) analysis revealed the decrease in the number of apoptotic hepatocytes in the eckol-treated group as compared to the CCl_4_ model group. Eckol enhanced the expression of Bcl-2 and suppressed the expression of cleaved caspase-3, which reduced malondialdehyde formation, enhanced superoxide dismutase, glutathione peroxidase activity, and glutathione content, elevated TNF-α, and suppressed IL-1β and IL-6. Eckol also increased the level of IL-10, an anti-inflammatory cytokine with the recruitment of CD11c^+^ dendritic cells into the liver tissue of CCl_4_-treated mice. Thus, inhibiton of apoptosis, oxidation, and inflammation including immune regulation are the multiple mechanisms related to protective effects of eckol [48].

In another study, hepatotoxicity in primary rat hepatocytes induced by doxorubicin was used to evaluate the hepatoprotective effects of 14 edible varieties of Korean seaweed. The *E. stolonifera* isolated phlorotannins dioxinodehydroeckol, eckol, phlorofucofuroeckol-A, dieckol, and triphlorethol A exhibited hepatoprotection with EC_50_ values of 3.4, 8.3, 4.4, 5.5, and 11.5 μg/mL, respectively. The importance of molecular weight regarding the hepatoprotective effects against doxorubicin-induced cytotoxicity was explained by the fact that phloroglucinol (molecular weight = 126) having a low molecular weight exhibited no effect, unlike the high molecular weight phlorotannins eckol and dieckol. In conclusion, eckol is suitable as potential therapeutics for the treatment of hepatotoxicity as compared to other evalauted phlorotannins [25].

Furthermore, the active EtOAc-soluble fraction of *E. stolonifera* presented phlorotannins (phloroglucinol, dioxinodehydroeckol, eckol, phlorofucofuroeckol-A, and dieckol) through bioassay-guided fractionation that was determined to protect against cytotoxicity in the human liver-derived HepG2 cells induced by tacrine [47]. Except for dioxinodehydroeckol and phlorofucofuroeckol-A exhibiting EC_50_ values of 62.0 and 79.2 µg/mL, respectively, other compounds, including eckol (EC_50_ > 100), exhibited no activity in HepG2 cells against the cytotoxic effects of tacrine [92].

### 2.4. Neuroprotective Activity

The age-dependent disorders which include neurodegenerative diseases are prevailing, as people are living longer than they did in the past [93]. Alzheimer’s disease (AD) is a major neurodegenerative disorder characterized by an increase in acetylcholinesterase (AChE) levels around β-amyloid plaques and neurofibrillary tangles [94]. Adverse intra- and extracellular effects of toxic α-synuclein and other Lewy body pathologies [95,96] lead to another prevalent neurodegenerative disorder known as Parkinson’s disease (PD). Due to the severe side effects of synthetic neuroprotective agents, there is a growing interest in nutraceuticals and other herbal alternatives [30]. 

In search of an anti-AD treatment derived from marine plants, the evaluation of the *E. bicyclis* and its phlorotannins against β-secretase 1 (BACE1) was carried out. Eckol (IC_50_ 12.20 µM) isolated from EtOAc soluble fraction showed significant activity against BACE1 [51]. Taking into account their potent anti-BACE1 activity, acetylcholinesterase (AChE) and butyryl cholinesterase (BChE) inhibitory assays on ethanolic extracts of 27 Korean seaweeds were evaluated. The ethanolic extract of *E. stolonifera* yielded *n*-hexane fraction containing sterols, fucosterol, and 24-hydroperoxy 24-vinylcholesterol, and the EtOAc fraction containing phlorotannins, phloroglucinol, dioxinodehydroeckol, eckol, phlorofucofuroeckol-A, dieckol, triphlorethol-A, 2-phloroeckol, and 7-phloroeckol, where eckol exhibited significant AChE inhibition with an IC_50_ value of 20.56 ± 5.61 µM. Conversely, eckol exhibited no inhibitory activity against BChE, with IC_50_ values > 500 µM. This difference in activity can be explained by the fact that AChE is a substrate-specific enzyme in nerve synapse, whereas BChE is a non-specific enzyme in plasma and tissue showing involvement of specific binding properties of enzyme and substrate [52].

Similarly, in a study related to *E. maxima* and its crude extract, solvent-soluble fractions, and isolated phlorotannins, the IC_50_ values of the solvent soluble fractions ranged from 62.61 to 150.8 μg/mL, with the EtOAc fraction having an excellent inhibitory activity against AChE. Compounds isolated from the EtOAc fraction were identified as phloroglucinol and two of its derivatives, dibenzo-1,4-dioxine 2,4,7,9-tetraol and eckol. Dibenzo-1,4-dioxine-2,4,7,9-tetraol (IC_50_ 84.48 ± 0.26 µM) and eckol (IC_50_ 76.70 ± 0.35 µM) exhibited better inhibitory activity against AChE than phloroglucinol (IC_50_ 579.32 ± 0.34 µM), suggesting that dibenzo-1,4-dioxine-2,4,7,9-tetraol and eckol could be effective as AChE inhibitors in treatment of AD [26].

Since the inhibition of monoamine oxidase (MAO) is important in the management of PD, eckol and dieckol isolated from methanolic extract of *E. bicyclis* were investigated for their effectiveness on their degree of human (*h)* MAO-A and *h*MAO-B inhibition. Eckol exhibited IC_50_ values of 7.20 ± 0.71 µM and 83.44 ± 1.48 µM against *h*MAO-A and *h*MAO-B, respectively. Mixed and non-competitive inhibitory mechanisms were revealed by biochemical and molecular docking examinations. Hence, eckol can be useful to manage PD and other neurological disorders through the inhibition of MAO [53].

Insomnia is currently a widespread sleep disorder worldwide leading to an increase in the use of natural sleep aids containing specific components of foods and herbal plants. Thus, these are becoming popular to enhance sleep quality and refrain side effects as compared to prescription sedative-hypnotics [97]. Gamma-aminobutyric acid (GABA)_A_–benzodiazepine (BZD) receptor, a well-recognized target for sedative-hypnotics was used to test the binding activity of methanol extracts of 30 seaweeds. Among the seaweeds, the most active ethanol extract of *E. cava* significantly increased sleep in mice when induced by pentobarbital. Among the four separated solvent fractions, hypnotic activity was related to the content of total phenols and total phlorotannins in the seaweed. The highest activity was exhibited by the EtOAc fraction which constituted of phlorotannins eckol, dioxinodehydroeckol, dieckol, and triphlorethol-A. Eckol (*K_i_* 1.070 µM) and dioxinodehydroeckol (*K_i_* 1.491 µM) showed better binding at the receptor than triphlorethol-A (*K_i_* 4.419 µM) and dieckol (*K_i_* 3.072 µM). The hypnotic effects of the ethanol extract and its EtOAc fraction were inhibited by flumazenil which is a specific GABA_A_–BZD receptor antagonist. Hence, through the positive allosteric modulation of the GABA_A_–BZD receptor, the phlorotannins from EC, including eckol, induces sleep [55].

The involvement of G-protein coupled receptors in human pathophysiology and their pharmacological traceability has emphasized on design and implementation of high-throughput G protein coupled receptor functional assays to identify novel drug candidates [98]. Dopamine receptors are specific therapeutic targets for PD, schizophrenia, and drug abuse. According to the stimulatory or inhibitory properties of the secondary messenger, cyclic adenosine monophosphate, dopamine receptors are categorized as Gα_s/olf_-coupled D_1_-like (D_1_ and D_5_) and Gα_i/o_-coupled D_2_-like (D_2_, D_3_, and D_4_) receptors [99]. At a concentration of 25 μM, eckol conferred a 10.60% and 36.55% of the control agonist effect on the human dopamine D_3_ receptor (*h*D_3_R) and human D_4_ receptor (*h*D_4_R), respectively. At 50 μM eckol, the agonist response increased to above 50%, giving EC_50_ values of 48.62 ± 3.21 and 42.55 ± 2.54 μM for *h*D_3_R and *h*D_4_R, respectively. However, eckol did not show any modulating effect on dopamine D_1_, serotonin _1A_ (5-HT_1A_), vasopressin (V_1A_), tachykinin (NK_1_) or muscarinic (M_5_) receptors [56]. In the context of molecular docking, negative binding energy (−6.41 kcal/mol) was exhibited by eckol, bound to the active site of the *h*D_3_R receptor, forming five H–bond interactions. Similarly, eckol bound to the active site cavity of *h*D_4_R with a negative binding energy (−6.46 kcal/mol) that was lower than dopamine (−5.68 kcal/mol), possibly as a result of four H–bond interactions. Even though eckol did not form a salt bridge to the carboxylate group of Asp110 of *h*D_3_R and Asp115 of *h*D_4_R important for high-affinity ligand binding to dopaminergic receptors [100], it formed an H–bond (O–H) interaction. Molecular dynamics (MD) simulation showed that the interaction of eckol with the binding pocket had changed significantly. Through the H–bonds, seven water molecules were involved in the interaction with eckol. In the MD simulation, Ser192 and Phe346 were the two new interacting residues. Eckol formed an H-bond interaction with Ser192 at a distance of 2.82 Å that was important for the activation of D_3_R. On the other hand, Phe346 displayed a hydrophobic interaction with eckol initiating a conformational change in the protein and ligand inside the binding pocket. Through π–π interactions, Phe346 also bound to eticlopride and dopamine contributing to the stabilization of ligand inside the binding pocket. The conserved serine residues in helix V also plays an important role as a molecular determinant for agonist-induced signaling from dopamine receptors. The Ser192 of *h*D_3_R (Ser197 of *h*D_4_R) and a hydroxyl moiety of eckol presented an H–bond interaction, implying eckol as a dual *h*D_3_/D_4_R agonist. A logP_o/w_ value of 2.99, an excellent binding to plasma protein (100%) and a moderate human intestinal absorption of 55.60%, was predicted through in silico pharmacokinetic parameter PreADMET. Also, the in vivo blood–brain barrier penetration calculations showed a moderate absorption (25%) by the central nervous system. [56]. 

### 2.5. Radioprotective Activity

Although ionizing radiation can have health benefits as radiation therapy in the treatment of cancer or thyrotoxicosis, it is often narrowed down by the toxicity to the neighboring normal tissues and organs [101,102]. Natural plant extracts have significant potential as adjuncts to successful radiotherapy [103] leading to further research on finding safer, less expensive, and more potent radioprotectors.

It was ascertained that eckol at doses of 10 mg/kg body weight given 2 h and 18 h before exposure to 8, 9 or 10 Gy of whole-body irradiation (WBI) could save mice from radiation (^60^Co gamma-ray)-induced damage. Representing a significantly diminished death rate, 28.6% (4/14) of the irradiated, untreated group survived for 30 days after exposure to 9 Gy of WBI while 86.6% (13/15) of the mice survived for 30 days that received eckol plus irradiation. An endogenous colony-forming unit (CFU) assay revealed 2.3 ± 0.5 and 3.5 ± 0.5 endogenous CFUs from the eckol plus irradiation group and irradiation control group, respectively. The results did not alter for the non-irradiated group when treated with eckol alone. The revival of hematopoietic ability by the splenic progenitor cells due to the eckol was indicated by the 50% increase in the number of CFUs. A decrease in the percentage of tail DNA (14.7 ± 5.6%) after eckol treatment in irradiation-damaged lymphocytes as compared to the content of tail DNA (42.5 ± 8.7%) when exposed only to 7 Gy of WBI was shown by alkaline comet assay. Thymidine incorporation of splenocytes increased by two fold in 9 days in eckol-treated irradiated mice as compared to the untreated one. Furthermore, the number of CD^3+^ pan T cells and CD45R/B220+ pan B elevated in the eckol-treated irradiated groups as compared with the irradiated, untreated control groups. The increase in the number of CD3^+^T by 44.3% and CD45R/B220^+^ pan B cell population by 27.6% as compared with the irradiated, untreated controls confirmed the role of eckol in immunoprotection by elevating the revival rates of specific immune cells [62].

Eckol and dieckol were also examined for their radioprotective activity in intestinal stem cells. Pretreating gamma-irradiated mice (3.5 days irradiation (8 Gy)), with phloroglucinol (crypts per circumference 43.73 ± 15.87) and eckol (crypts per circumference 32.08 ± 17.96) remarkably increased the jejunal villi height (*n* = 6 per group) compared with the irradiated controls (crypts per circumference 8.6 ± 1.7). The number of surviving crypts also increased in gamma-irradiated mice (3.5 days irradiation (8 Gy)) pretreated with phloroglucinol and eckol as compared to the irradiated control, suggesting an improvement in the survival of the jejunal crypt in irradiated mice when treated with these compounds. The expression of apoptotic nuclei (occurring mainly in the lower third of the crypt) was increased by irradiation as shown by both hematoxylin–eosin staining and the in situ DNA end- labeling (ISEL) method. A decline in the number of apoptotic nuclei was observed 12 h after irradiation when pretreated with phloroglucinol and eckol as compared with irradiated control groups. Thus, improvement in jejunal crypt survival and shielding against apoptosis induced by radiation in mouse jejunal crypts was observed when pretreated with phloroglucinol and eckol [63].

To further support the radioprotective potential of eckol, *E. cava* was evaluated to determine its cyto- and histoprotective ability in lymphocytes and the intestine against damage induced by whole body irradiation (WBI) in vivo. To assess whether the effectiveness of eckol in inhibiting DNA damage was accountable to apoptotic changes, the nuclear morphology of lymphocytes was assessed in irradiated mice as peripheral blood lymphocytes are easily harmed by ionizing radiation. The number of apoptotic nuclei was reduced dramatically in the eckol treated group in comparison with the irradiated control group (9.95 ± 0.50% versus 14.85 ± 0.39%). Hence, the viability of peripheral blood lymphocyte was protected by inhibiting apoptotic cell death induced by ionizing radiation in vivo. The frequency of apoptotic fragments in crypt cells was reduced in the eckol-treated group by 16.63% compared to the untreated group (4.51 ± 0.30%) after 24 h of WBI treatment revealing the ability of eckol to protect intestinal crypt cells from radiation-induced apoptosis. The increase in the expression level of p53 and Bax, commonly seen after WBI, was also reduced by eckol with the induction in the expression level of Bcl-2, which is involved in apoptosis and DNA repair. In those ways, eckol treatment modulated the immunohistochemical localization and magnitude of apoptosis-related proteins in jejunal crypt cells. Therefore, eckol administration could provide great benefits for cancer patients by effectively preventing the apoptosis of peripheral blood lymphocytes and intestinal crypt cells induced by radiation [62]. Furthermore, eckol pre-treatment scavenged ROS in γ-irradiated V79-4 cells and protected against DNA damage by decreasing 8-OHdG adduct of DNA, a biomarker of oxidative stress. Eckol also decreased the lipid peroxidation in the γ-irradiated V79-4 cells. The apoptosis induced by γ-irradiation was also decreased in eckol (10 μg/ml) pre-treated cells with a decrease in DNA fragmentation. An increase in Bcl-2 expression and a decrease of Bax expression in γ-ray irradiated cells treated with eckol further inhibiting the caspase-dependent pathway via mitochondria. The SEK1-JNK-AP-1 is the major pathway that is suppressed by eckol for decreasing the γ-ray irradiation-induced apoptosis [65]. 

### 2.6. Anti-Photo Aging Effects

In addition to the human process of aging, the areas of the face, neck, and dorsum of hands and forearm get affected by the harmful effects of UV light. Photo aging results due to the continued UV exposure and sun damage on human aging skin [104] leading to the disorganization in the collagen fibrils and the aggregation of atypical, amorphous, elastin-containing material [105]. Photo aging is responsible for the undesirable age-associated transformation in the skin, including coarseness, wrinkling, sallow color, telangiectasia, and an array of neoplasms [106].

In matrix metalloproteinase (MMP) family, the expression of certain members is induced by UV irradiation. Matrix metalloproteinases are zinc-containing proteinases that degrade collagen and the other extracellular matrix proteins comprising the dermal connective tissue [107,108,109]. Proteolytic events in the human system which include embryogenesis, wound healing, inflammation, angiogenesis, and cancer involve MMPs [109,110,111,112]. The connective tissue damage that occurs in photo aging is due to MMP-mediated collagen destruction [113], where MMP-1 is the major factor involved in the deterioration of dermal collagen in the process of aging [114,115]. Evaluation of 29 seaweed extracts for active compounds that can inhibit MMP-1 expression led to seaweeds *E. stolonifera, E. bicyclis*, and *E. cava* exhibiting more potent inhibition of NF-κB and AP-1 than seaweeds from other families. They also showed protection against collagen destruction through the TNFα-induced inhibition of MMP-1 expression. Eckol and dieckol isolated from *E. stolonifera* diminished the expression of MMP-1 induced by TNF-α and basal expression of MMP-1, and reduced both NF-κB and AP-1 reporter gene activity in human dermal fibroblasts. Hence, these results indicate that the reduction of MMP-1 expression is due to the compounds eckol and dieckol [57].

The excessive generation of ROS causing oxidative stress in the skin is due to the UVB radiation ultimately damaging DNA, leading to cancer [116,117]. According to studies on skin cells, it has been demonstrated that the damage of molecules and alteration of their structures, resulting in changes in cellular functions is also due to UVB radiation. Insights were reported about eckol and its ability to protect against UVB-induced oxidative cell damage in human skin keratinocytes (HaCaT). Eckol decreased the UVB-induced levels of intracellular ROS eventually lowering the damage to cellular components caused by oxidative stress. The cell viability increased by 16% in UVB-irradiated cells treated with eckol (83%) as compared to UVB irradiated cells without eckol (67%) [58].

The desirability of developing effective chemo-preventive agents against UVB-induced skin cancer in humans has led to an assessment of the chemo-preventive efficacy of brown algae polyphenols (2-*O*-(2,4,6-trihydroxyphenyl)-6,6´-bieckol, 6,6´-bieckol, 8,8´-bieckol, 7-phloroeckol, 2-phloroeckol, eckol, dieckol, phlorofucofuroeckol, phlorotannin A, fucofuroeckol A, and various minor homologues) against photo carcinogenesis in the SKH-1 hairless mouse skin model. A decrease in tumor incidence was seen when brown algal polyphenols were administered through diet (79%) and skin (94%) with moderate protection against skin carcinogenesis induced by UV-B radiation in mice. The propagation of tumor decreased from 8.54 ± 0.75 to 4.73 ± 0.74 (*p* < 0.005) and 3.75 ± 0.54 (*p* < 0.001) when treated with 0.1% and 0.5% brown polyphenols through diet, in tumors/tumor-bearing mice, exhibiting a 45% and 56% inhibition. On the other hand, topical administration of 3 and 6 mg of these compounds reduced tumor multiplicity from 8.45 ± 1.23 to 3.42 ± 0.56 (*p* < 0.001) and 4.56 ± 0.56 (*p* < 0.005), respectively, in tumors/tumor-bearing mice. They also suppressed the gene expression of cyclooxygenase (COX)-2, decreased PGE_2_ levels in the skin, inhibited the amount of COX-2 protein, and reduced the rate of proliferating cells in the epidermis. In summary, eckol exerts protective effects against skin carcinogenesis induced by UVB radiation [59]. Also in another study, the photo-protective effects of phlorotannins on the cell damage caused by UVB radiation was demonstrated. The *E. cava*-isolated phlorotannins (phloroglucinol, eckol, and dieckol) and polyphenol were examined for their protective effect against UVB-induced photo-oxidative stress in human fibroblast cells. Eckol exhibited slightly low activity against UVB radiation-mediated ROS (174.4% ROS at 250 µM) as compared to dieckol (100.7% ROS at 250 μM) when the level of ROS was recorded as 234.1% in UVB-irradiated cells (positive control). Eckol showed cell survival rates of 45%, 50%, and 56% at concentrations of 5, 50, and 100 µM, respectively. The comet assay was performed to evaluate whether phlorotannins protected against DNA damage and revealed that eckol had about 21%, 43%, and 53% inhibitory activity at concentrations of 0.5, 5, and 50 µM, respectively [60].

### 2.7. Anti-Hypertensive Activity

The regulation of blood pressure involves angiotensin-converting enzyme (ACE), a potent vasoconstrictor, whose role is to convert angiotensin I to angiotensin II. Hence, ACE inhibition plays a physiological role in the treatment of hypertension. Natural ACE inhibitors as alternatives with fewer side effects as compared to synthetic drugs have been a major interest for research in preventing hypertension [118]. Among the ethanol, EtOAc, chloroform, hexane, and diethyl ether extracts of *E. cava*, the strongest ACE inhibitory activity was demonstrated by the ethanol extract (IC_50_ 0.96 mg/mL). The ethanol extract consisted of phlorotannins which exhibited potent ACE inhibition: phloroglucinol (IC_50_ 2.57 ± 0.09 mM), triphlorethol-A (IC_50_ 2.01 ± 0.36 mM), eckol (IC_50_ 2.27 ± 0.08 mM), dieckol (IC_50_ 1.47 ± 0.04 mM), and dioxinodehydroeckol (IC_50_ 2.95 ± 0.28 mM). Eckol showed lower IC_50_ than that of the weakest dioxinodehydroeckol. The protein-binding abilities of the phlorotannins occurring reversibly by hydrogen bonding or irreversibly through covalent condensations could be associated with the ACE inhibition [119]. 

Similarly, significant inhibition of more than 50% at a concentration of 163.93 μg/mL was exhibited by the ethanol extracts of seaweeds which included *E. stolonifera, E. cava, Pelvetia siliquosa, Undaria pinnatifida*, and *Grindelia tenella.* Since the marked ACE inhibitory activity was exhibited by crude extract of *E. stolonifera,* it was fractionated with several solvents in order to determine the ACE inhibitory properties between the concentrations of 32.69 and 169.72 μg/mL. Fucosterol and six phlorotannins (phloroglucinol, dioxinodehydroeckol, eckol, phlorofucofuroeckol-A, dieckol, and triphlorethol-A) were successively isolated from the EtOAc fraction. Eckol, phlorofucofuroeckol-A, and dieckol displayed IC_50_ values of 70.82 ± 0.25, 12.74 ± 0.15, and 34.25 ± 3.56 µM, respectively, showing the importance of a closed-ring dibenzo-1,4-dioxin moiety to the observed ACE-inhibition [28]. 

### 2.8. Anticoagulative Activity

Anticoagulants, commonly described as blood thinners, inhibit thrombin and factor Xa (serine proteases), which is promoted by stimulating the activity of serine protease inhibitor, SERPIN-antithrombin III. Difficulty in isolating heparin (primary anticoagulant) and its hemorrhagic side effects have driven researchers to isolate novel anticoagulants from natural sources without side effects [31].

Eckol was isolated from *Ecklonia kurome* to evaluate the structural requirements necessary for inhibition of α_2_ macroglobulin (α_2_-M) and α_2_ plasmin (α_2_-P) inhibitors. The main plasmin inhibitors in plasma include the α_2_-M and α_2_-P inhibitors. Eckol exhibited potent and specific inhibitory activity on the actions of α_2_-M (IC_50_ 2.5 µg/mL) and α_2_-P (IC_50_ 1.6 µg/mL). The synthesis of various methylated derivatives of eckol in this study and their inhibitory activity on the anti-plasmins implied that a simple dibenzo-1,4-dioxin skeleton bearing some functional groups could become a lead compound in the development of a new class of thrombolytic agents [120].

In a similar study, eckol acted as an α_2_-P (IC_50_ 1.6 µg/mL), an α_2_-M (IC_50_ 1.8 µg/mL), an α_1_ anti-trypsin (IC_50_ 0.8 µg/mL), and thrombin (IC_50_ 13 µg/mL) inhibitor. However, eckol exhibited very weak inhibition on anti-thrombin III-heparin complex and exhibited no inhibition on plasmin. The inhibitory activity of eckol was reduced in whole human plasma, but at a concentration above 200 μg/mL, it enhanced urokinase-induced fibrinolysis. The importance of dibenzo-1,4 dioxane skeleton for inhibition of plasmin inhibitors was shown by several derivatives of eckol [16]. As previously stated [120], only the dibenzo-1,4-dioxane skeleton (dibenzo-*p*-dioxin-1,3,6,8-tetraol) is important for the potent anti-inhibitor activity as compared to the other hydroxyl groups present in eckol. Hence, the results suggest that a simple dibenzo-1,4-dioxane skeleton with other functional groups could be clinically useful in stimulating fibrinolysis [16].

### 2.9. Antibacterial and Antiviral Activity

Despite the boost in the expansion of antibacterial agents, the development of multidrug-resistant bacteria has created a special need for research on new antibacterial agents [121]. As the need grows, the development of more powerful natural antimicrobial agents with minimal side effects and toxicity is a necessity for public health [122]. A study designed to evaluate the combined activity of ampicillin and eckol against methicillin-resistant *Staphylococcus aureus* (MRSA) and *Salmonella* isolates revealed the antimicrobial activity of eckol towards all the tested strains. The minimum inhibitory concentrations (MICs) of eckol towards *S. aureus* and *Salmonella* showed inhibition in a concentration-dependent manner, but all concentrations caused a reduction in CFUs over time, showing at least a 4 log10 decrease in the bacterial count after 4 h. Concentrations between 125 and 500 µg/mL showed bacteriostatic activity while that between 250 and 500 µg/mL inhibited the expansion of *Salmonella* for a period of 24 h. The fraction inhibitory concentration index ranged from 0.31 to 0.5 for the combination of eckol and ampicillin, showing a high inhibition against *S. aureus* when ampicillin was used in combination with eckol as compared to when it was used alone [29].

The EtOAc-soluble extract of fermentation broth of *E. bicyclis* with *Candida utilis* YM-1, which included eckol, dieckol, dioxinodehydroeckol, and phlorofucofuroeckol-A exhibited a strong anti-MRSA activity, with MIC values in the range of 128 to 512 μg/mL indicating a potent antimicrobial inhibition of MRSA and foodborne pathogenic bacteria [123]. 

Also, eckol exhibited MIC values of 256 μg/mL for *Propionibacterium acnes* (KCTC 3314), *P. acnes* isolate 2875, *P. acnes* isolate 2876, and *Staphylococcus aureus* (KCTC 1927), and a MIC value of 128 μg/mL for *Staphylococcus epidermidis* (KCTC 1370) [124].

In the viral lifecycle, neuraminidase (NA) plays a critical role as a target in the development of drugs against influenza. A high NA-inhibitory activity (72.1% inhibition at 30 μg/mL) of EtOAc layer of ethanol extract of *E. cava* led to bioactivity-guided fractionation and isolation of five phlorotannins (phloroglucinol, eckol, 7-phloroeckol, phlorofucofuroeckol, and dieckol). The inhibitory activity and kinetic behavior of these phlorotannins were examined against NA using various strains of influenza virus which includes A/Bervig_Mission/1/18 (H1N1), A/PR/8/34 (H1N1), A/Hong Kong/8/68 (H3N2), and A/Chicken/Korea/MS96/96 (H9N2). Eckol demonstrated a moderate IC_50_ value of 89.5 µM against A/Bervig_Mission/1/18 (H1N1), but failed to show any significant inhibition on the other virus strains (IC_50_ > 200 µM) compared with the other tested compounds (7-phloroeckol, phlorofucofuroeckol, and dieckol). The IC_50_ of the compounds increased with the increase in hydroxyl groups (eckol to dieckol), indicating the significant and definite NA inhibitory activity due to the number of hydroxyl groups on the structure of phlorotannins [66]. 

Similarly, viral hemorrhagic septicemia virus (VHSV) in the fathead minnow (FHM) cell line was used to test the inhibition efficacy of *E. cava* extract and its compounds. At low concentrations of 10 μg/mL, the extract, eckol, and phlorofucofuroeckol-A exhibited potent virus inhibitory activity. When there was simultaneous co-exposure of VHSV to extract, eckol, and phlorofucofuroeckol-A in the FHM, the inhibition rates increased (46.4–96.4%) as compared to the pre- (16.5–48.4%) and post-exposure (39.5–56.5%) strategies with the EC_50_ values of 4.76, 1.97, and 0.99 μg/mL for the extract, eckol, and phlorofucofuroeckol-A, respectively. Also, an increase in the exposure time led to high activity against the virus in the FHM cell line. After the oral administration of the extract to the olive flounder challenged with VHSV, the efficacy against VHSV resulted according to the VHSV challenge concentration and the dose of extract. The increase in proinflammatory cytokines (IL-1β, IL-6, and IFN-γ) expression and the activation of IFN-α/β, ISG15, and Mx indicated the immunomodulatory and immunostimulatory effect of the extract [67]. 

Human immunodeficiency virus (HIV) is a retrovirus affecting the cells of the human immune system and infecting the vital organs, ultimately causing immune deficiency and leading to acquired immune deficiency syndrome (AIDS). As there is an increase in failure in anti-AIDS treatment due to the strains of the virus becoming drug-resistant, the research for drug candidates with higher rates of inhibition against various strains of HIV is increasing with the major focus on naturally derived compounds and their derivatives. The bioassay-directed isolation of *E. cava* led to the isolation of eckol, 8,8’-bieckol, 8,4’dieckol, and phlorofucofuroeckol-A. 8,8´-bieckol, 8,4´dieckol inhibited HIV-1 reverse transcriptase (RT), and protease, whereas eckol and phlorofucofuroeckol-A failed to show any activity, even if they were produced from RT inhibitor fractions having ≥80% inhibition (100 mg/mL) [125].

Given the importance of severe acute respiratory syndrome coronavirus 3C-like proteinase (SARS-CoV 3CL^pro^) in viral replication, a biological assessment was completed on the phlorotannins isolated from *E. cava*. In this study, the *E. cava* phlorotannins were shown to inhibit SARS-CoV3CL^pro^ competitively in cell-free systems. Phloroglucinol, triphlorethol A, eckol, dioxinodehydroeckol, 2-phloroeckol, 7-phloroeckol, fucodiphloroethol G, dieckol, and phlorofucofuroeckol-A were isolated from the ethanol extract of *E. cava*. Except for phloroglucinol, all the other phlorotannins displayed inhibition on 3CL^pro^ hydrolysis in a dose-dependent manner. The IC_50_ values ranged from 2.7 to 164.7 µM where eckol (IC_50_ 8.8 µM) and dieckol (IC_50_ 2.7 µM) exhibited potent inhibitory effect on SARS-CoV 3CL^pro^ cell-free cleavage with eckol structure being essential and crucial for good inhibition of SARS-CoV 3CL^pro^ [68].

### 2.10. Anti-Obesity Activity

High food intake in the absence of exercise leads to obesity [126,127] contributing to the growth in metabolic syndrome, diabetes mellitus, atherosclerosis, osteoarthritis, cardiovascular diseases, and non-alcoholic fatty liver disease [127,128,129]. In order to investigate the significance of *E. cava* on body weight, fat content, and hyperglycemia in obese mice, they were fed with extracts (200 mg/kg) of *E. cava* collected from Jeju (J-CA) or Gijang (G-CA) after 3 weeks of high-fat diet (HFD) induction. High- performance liquid chromatography revealed both G-CA (4.72 mg/g) and J-CA (12.98 mg/g) to be rich in eckol. The G-CA-HFD mice showed lower body weight and little weight gain (40% decrease), reduced ALT and cholesterol levels, significantly increased mRNA levels of peroxisome proliferator-activated receptor (PPAR)γ2, C/EBPα and sterol regulatory element-binding proteins (SREBP)-1C (adipogenic-related transcription factors), higher fatty acid synthase (FAS) as compared to PBS-HFD mice and J-CA-HFD mice, whereas the plasma glucose level showed a decrease in J-CA-HFD and G-CA-HFD as compared to PBS-HFD. Due to the relationship between obesity and T2DM with chronic inflammation, the expression of inflammatory cytokines, TNF-α, interleukin (IL)-1β, and the macrophage marker F4/80 was elevated in PBS-HFD mice as compared with the normal chow mice. The G-CA-HFD mice significantly decreased all the above inflammatory factors as compared to PBS-HFD mice. The increase in hepatic lipid while HFD feeding caused the development of non-alcoholic fatty liver conditions. Therefore, the lipid content in frozen liver sections was assessed with an obvious increase in lipid droplet accumulation in the PBS-HFD mice. In comparison to PBS-HFD mice, there was a decrease in lipid droplet and triglyceride content in G-CA-HFD and J-CA-FD mice. Also, the mRNA expression of acetyl-CoA carboxylase 1 (ACC1), FAS, and SREBP-1c (genes related to lipid metabolism) significantly decreased in the G-CA-HFD mice in comparison to the PBS-HFD mice [78]. 

Obesity is related to adipogenesis which is the process of pre-adipocyte differentiation into adipocytes due to the high energy input and lack of exercise. Adipogenesis is regulated by multiple processes, including pre-adipocyte proliferation, differentiation, and fatty acid oxidation and synthesis controlled by several factors. In one of the study, the inhibitory effects of enzyme-treated *E. cava* extract containing three distinct phlorotannins (eckol, dieckol, and phlorofucofuroeckol-A), separated and purified by pectinase and cellulose, was examined on the adipogenesis of 3T3-L1 adipocytes. When 12.5, 50, and 200 µg/mL of extract was applied to the cells for 24 to 48 hours, the cell viability increased by 49.8%, 58.3%, and 65.5%, respectively; the glucose utilization decreased by 143.5 ± 14.6, 138.9 ± 7.3, and 96.7 ± 6.2 mg/dL, respectively; and triglyceride accumulation also decreased by 143.5 ± 8.7, 92.4 ± 1.3, and 77.1 ± 1.3 mg/dL, respectively. Treatment with 12.5, 50, and 200 µg/mL extract also decreased the lipid accumulation by 52.2%, 53.2%, and 62.6%, respectively in 3T3-L1 adipocytes, including a decrease in the expression of CCAAT-enhancer-binding proteins (C/EBP)β but not C/EBPδ or PPARγ. A decrease in the protein levels of SREBP-1c, adipocyte fatty acid binding protein, FAS, and adiponectin was also observed [27].

Among the various potential targets for treating obesity in humans, pancreatic lipase holds great potential. Pancreatic lipase is involved in the absorption of triglycerides by the small intestine which eventually gets secreted by the pancreas. It is responsible for the hydrolysis of triglycerides into glycerol and fatty acids [130]. An investigation of a methanolic extract of *E. bicyclis* revealed significant inhibition on pancreatic lipase through porcine pancreatic lipase assay (IC_50_ value 36.4 ± 2.9 mg/mL) leading to the isolation of eckol, fucofuroeckol A, 7-phloroeckol, dioxinodehydroeckol, phlorofucofuroeckol-A, and dieckol. Since eckol lacks ether linkage at the C-7 position of its phlorotannin skeleton, it exhibited much weaker activity against pancreatic lipase than fucofuroeckol A and 7-phloroeckol, having one phloroglucinol element in the same position. Also, the molecular weight difference between the compounds had no impact on their pancreatic lipase inhibitory activity. The existence of a phloroglucinol moiety in eckol structure at the C-7 position and the number of hydroxyl groups in eckol, fucofuroeckol A, and 7-phloroeckol might have positively influenced the inhibition of pancreatic lipase, probably through their unique receptor binding [79]. 

### 2.11. Anticancer and Cytotoxic Activity

The abnormal cell growth leading to invasion or spreading throughout the body can be defined as cancer. External factors (tobacco, alcohol, chemicals, infectious agents, and radiation) and internal factors (hormones, immune conditions, inherited mutations, and mutations occurring during metabolism) trigger the mutation in chromosomal DNA of normal cells, eventually resulting to cancer [131,132]. Although there is the availability of anti-neoplastic drugs and chemotherapy, the detrimental aftermath has sparked a search for natural products that could be used as new therapeutic agents.

As eckol exhibited a wide range of therapeutic activities, it was also tested against cancer. When treated with eckol, the glioma stem-like cell markers and self-renewal-related proteins changed their expressions, their ability to form spheres and anticancer treatment sensitivity. Eckol treatment effectively reduced the sphere formation, CD133^+^ cell population and concealed the expression of glioma stem-like cell markers and self-renewal-related proteins in the absence of cell death.The fusion of eckol with glioma stem-like cells diminished tumor formation and the anchorage-independent growth of cells on soft agar in xenograft mice. Eckol decreased the resistance of glioma stem-like cells to ionizing radiation and temozolomide. In addition, both the PI3K-Akt and Ras-Raf-1-Erk signaling pathways were blocked due to the eckol treatment in the glioma stem-like cell. Hence, eckol suppresses stemness and malignancies, and sensitizes glioma-stem-like cells to anticancer treatments, providing a novel therapeutic strategy specifically targeting these cells [75].

In another study related to anti-cancer effects, four phlorotannins and fucosterol were isolated and characterized from *E. maxima*. The compounds were phloroglucinol, eckol, 7-phloroeckol, 2-phloroeckol, and fucosterol. The cytotoxic effects of the compounds were tested on a few cancer cell lines using the 3-(4,5-dimethylthiazol-2-yl)-2,5-diphenyl tetrazolium bromide (MTT) reduction assay. The results indicate that eckol had the highest activity against the selected cancer cell lines: HeLa cells, H157, and MCF7, making it a good candidate with prominent inhibitory activity against metastasis that could effectively reduce cell damage [76].

### 2.12. Anti-Histaminic Activity

The chemical or immunological stimulation of mast cells leads to the discharge of internal mediators, such as histamine, which is an indicator of cell degranulation, and diversity of several mediators of inflammation (eicosanoids, proteoglycans, proteases) including pro-inflammatory and chemotactic cytokines (TNF-α and ILs) leading to allergic diseases [133,134].

In one study, an in vitro assay related to hyaluronidase inhibition by phlorotannins was evaluated. The *E. bicyclis* and *E. kurome* phlorotannins exhibited potent inhibition in comparison to the popular inhibitors catechins and sodium cromoglycate. Phloroglucinol, eckol (trimer), phlorofucofuroeckol-A (pentamer), dieckol, 8,8’-bieckol (hexamers), and an unknown tetramer showed IC_50_ values of 280, >800, 140, 120, 40, and 650 µM, respectively, whereas catechin, epigallocatechin gallate, and sodium cromoglycate had an IC_50_ value of 620, 190, and 270 µM, respectively. According to the results, eckol (IC_50_ > 800 µM) showed no inhibitory effect on hyaluronidase activity [74]. 

Similarly, histamine release assay was performed on human basophilic leukemia (KU812) and RBL2H3 to assess the anti-allergic activities of phloroglucinol derivatives, fucodiphloroethol G, eckol, and phlorofucofuroeckol-A isolated from *E. cava*. Their inhibitory potential on the release of histamine was evaluated by their ability to inhibit immunoglobulin E (IgE) and anti-IgE antibodies stimulated degranulation of KU812 cells. Fucodiphloroethol G, eckol, and phlorofucofuroeckol-A displayed 23.97%, 44.26%, and 34.54% of relative histamine release, respectively, at the highest concentration (100 µM). In both the KU812 and RBL2H3 cells, calcium ionophore A23187 mediated the inhibitory effect of the phlorotannins. The histamine release percentages in RBL-2H3 cells treated with fucodiphloroethol G, eckol, and phlorofucofuroeckol-A were 27.73%, 50.63%, and 34.18%, and the degranulation percentages of RBL-2H3 cells were 18.02%, 31.67%, and 20.80%, respectively. Eckol, having fewer hydroxyl functional groups than the other two compounds, showed less inhibition on the histamine release in both cell lines, particularly compared with fucodiphloroethol G comprising of 11 hydroxyl functional groups. The molecular structure and amount of phenol groups could be the important factors for fucodiphloroethol G and phlorofucofuroeckol-A for showing strong activity than eckol. Also there was a potent inhibition of the binding activity between IgE and its receptor (30.58% and 34.23%) when treated with the highest concentration of fucodiphloroethol G and phlorofucofuroeckol-A, whereas eckol displayed less inhibiton (47.60%) in the flow cytometric analysis [73]. Thus, eckol from *E. cava* showed moderate antiallergic activity as compared to other compounds due to the structural differences amid the compounds in terms of the number of hydroxyl groups and the linkages among the phenol units, leading to different inhibitory activity. 

According to Sugiura et al. [19], the suppression of β-hexosaminidase release from RBL-2H3 cells by phlorotannins occurred according to their molecular size or number of phenol groups. Although eckol exhibited significantly lower activity than the positive control, epigallocatechin gallate (IC_50_ 22.0 µM), the other isolated phlorotannins 6,8´-bieckol, 6,6´-bieckol, phlorofucofuroeckol-B, and 8,8´-bieckol, phlorofucofuroeckol-A showed inhibitory activity similar to or greater than epigallocatechin gallate. In model rats fed with a diet including *Eisenia arborea*, the allergy symptoms improved possibly due to the suppressive effects of the isolated phlorotannins on enzyme and release of histamine. 

### 2.13. Anti-Inflammatory Activity

The occurrence of infection, endotoxin exposure, or cell injury in the body results in complex defensive feedback called inflammation that basically leads to the revival of normal cell structure and function [135]. There is a marked elevation in permeability to fluids and solutes due to the disturbance in the vascular barrier integrity, which is the central pathophysiologic mechanism of several vascular inflammatory diseases, such as sepsis and atherosclerosis [136,137,138]. Therefore, the change in permeability of the endothelial barrier contributes to the occurrence of anaphylaxis, sepsis, and acute lung injury [139,140].

Eckol and its derivatives were evaluated for their barrier protective effects against proinflammatory responses in human umbilical vein endothelial cells (HUVECs) and in mice. According to the results, the eckol and dieckol isolated from *E. bicyclis* showed inhibition of barrier disruption mediated by LPS and trans-endothelial migration of leukocytes to human endothelial cells at a concentration of 10 μM. In addition, eckol inhibited acetic acid-induced hyperpermeability and leukocyte migration induced by carboxymethylcellulose–sodium in vivo. Interestingly, according to the results obtained, the hydrogen donating hydroxyl groups present in dieckol regulated its protection ability which was better than eckol [69].

Similarly, the signaling pathways involved in the protective actions of eckol and dieckol, including phloroglucinol, against pro-inflammatory responses in HUVECs and in high-mobility group box 1 (HMGB1) protein-treated mice were investigated. These phlorotannins inhibited LPS (100 ng/mL)-induced HMGB1 release and the expression of Toll-like receptor 4. In addition, they also inhibited barrier disruption medidated by HMGB1, the expression of cell adhesion molecules (CAMs) (VCAM-1, ICAM-1 and E-selectin) and trans-endothelial migration of leukocytes to human endothelial cells. In addition, they suppressed hyperpermeability induced by acetic-acid and leukocyte migration induced by carboxymethylcellulose in vivo. To further explain the importance of hydroxyl group for the protective action, a change with the methyl group at the hydroxyl position of dieckol, diminished the vascular barrier protective effects [24]. 

The arachidonic acid (AA)-derived lipid-mediator secretory phospholipase A_2_s (sPLA_2_s) are esterases cleaving glycerophospholipids at the sn-2 position, ultimately liberating a fatty acid and a lysophospholipid [141] that are then present in synovial fluid, articular cartilage, and blood in patients suffering from rheumatic diseases [142]. Another lipid mediator derived from arachidonic acid that is involved in inflammation is biosynthesized by pathways that depend on COX and lipoxygenases (LOX) enzymes. An inhibitor of those enzymes could treat inflammatory diseases, atherosclerosis, and cancer. Five phlorotannins (i.e., phloroglucinol, eckol, dieckol, phlorofucofuroeckol-A, and 8,8’-bieckol) purified from *E. bicyclis* were evaluated for their potential inhibition on bee venom, porcine pancreas secretory phospholipase A_2_, soybean lipoxygenase, 5-LOX, COX-1, and COX-2. In the case of the bee venom sPLA_2_, the phlorotannins eckol, phlorofucofuroeckol-A, dieckol, 8,8´-bieckol, resveratrol, and epigallocatechin gallate demonstrated IC_50_ values of 120, 130, 160, 180, 170, and 110 μM, respectively. Eckol, phlorofucofuroeckol-A, dieckol and 8,8’-bieckol showed high activity in comparison to the positive controls resveratrol and epigallocatechin gallate. In porcine pancreas sPLA_2_, eckol, phlorofucofuroeckol-A, dieckol, and 8,8’-bieckol demostrated IC_50_ values of 100, 150, 90, and 180 μM, respectively_._ Eckol showed less inhibition against soybean lipoxygenase (LOX) and 5-LOX than 8,8´-bieckol, which was the potent inhibitor of soybean LOX, showing inhibition of more than 90% at 50 μM, and 5-LOX (IC_50_ 24 µM). The phlorotannins had negligible inhibitory effects on COX-1 and COX-2, with eckol exhibiting 43.2% inhibition of COX-2 at 100 μM [72].

In acne vulgaris, *P. acnes* bacteria [143] stimulates keratinocytes to activate TNF-α and ILs and other inflammatory mediators including nitric oxide secretion [144]. With an increase in MMPs, there is degradation of collagen in dermis infected with acne, which is predicted to form degraded/disintegrated collagen in the tissue [43]. The *P. acnes*-induced inflammatory response in HaCaT cells is due to MMP-2 and MMP-9 [145]. Eckol (1, 5, and 10 μM) slightly decreased the MMP-2 and MMP−9 mRNA levels. Also, *P. acnes* activated the NF-κB pathway, which is related to inflammatory mediators TNF-α, iNOS, and COX-2, and also the phosphorylation of p65 (an activated NF-κB subunit). Eckol gradually decreased all those activations in a concentration-dependent manner. Whereas treatment with *P. acnes* accelerated the phosphorylation of Akt which was inhibited by eckol in HaCaT cells [71].

In another study, arachidonic acid (AA), 12-*O*-tetradecanoylphorbol-13-acetate (TPA), and oxazolone (OXA) were used as three sensitizers for inducing ear edema of Institute of Cancer Research (ICR) mice. Eckol, 8,8’-bieckol, phlorofucofuroeckol-A, and phlorofucofuroeckol-B suppressed ear edema at a dose of 0.01 or 0.1 mg. The phlorotannins behaved more strongly towards ear edema when treated with AA and OXA rather than TPA. All the sensitizers were inhibited by the phlorotannins in a similar manner to epigallocatechin gallate (typical inhibitor) [23]. 

Following the above research, *E. arborea* purified six phlorotannins (6,8´-bieckol, 8,8´-bieckol, phlorofucofuroeckol-A, phlorofucofuroeckol-B, eckol, and 6,6´-bieckol,) were administered orally to mice initially, who were then evaluated for inhibitory effects of the compounds on swelling of the ears. To determine the suppressive ability of phlorotannins, a cultured mast cell model using RBL-2H3 cells was evaluated against the release of histamine, leukotriene B4, or prostaglandin E2 including their mRNA expression and the activity of COX-2. Eckol and 6,8′-bieckol suppressed degranulation at a concentration of 100 μM in RBL cells, compared with epigallocatechin gallate. As a result, all the phlorotannins showed potent inhibition with 6,8´-bieckol, 8,8´-bieckol, phlorofucofuroeckol-A having the highest activity [70].

### 2.14. Anti-Hyperlipidemic Activity

Increase in the level of lipids (fats, cholesterol, and triglycerides) in the blood leads to several acquired or genetic disorders referred to as hyperlipidemia. Cardiovascular or heart diseases are the major complications of hyperlipidemia prevented through the appropriate use of medications and a healthy lifestyle. In search of anti-hyperlipidemic agents, the effects of eckol and dieckol isolated from ethanolic extract of *E. stolonifera* were investigated on lipid levels in the serum of hyperlipidemic rats. A high-cholesterol diet or poloxamer 407 was used to induce hyperlipidemia in rats. The rats fed with a high-cholesterol diet showed elevated levels of TC (255.6 ± 8.7 mg/dL), TG (240.2 ± 14.0 mg/dL), and LDL-C (145.1 ± 10.4 mg/dL) in the serum, as well as an increased atherogenic index (A.I.) (3.47 ± 0.4). Eckol at a dose of 20 mg/kg of body weight once a day for 3 days, reduced the serum TC (157.0 ± 9.7 mg/dL), TG (174.9 ± 14.6 mg/dL), LDL-C (63.1 ± 10.1 mg/dL), and A. I. (1.77 ± 0.3). In addition, the HDL-C level of hyperlipidemic control rats induced by poloxamer 407 was 59.9 ± 5.6 mg/dL, and that of the eckol-treated rats increased to 66.2 ± 5.2 mg/dL [80].

Because the oxidation of LDL is one of the causative agent of atherosclerosis, the inhibitory effects of *E. stolonifera*, its fractions, the phlorotannins isolated from them, and the conjugated diene formation of the methanolic extract was investigated on in vitro Cu^2+^-induced human LDL oxidation. The thiobarbituric acid reactive substances (TBARS) assay revealed that the methanolic extract had concentration-dependent inhibitory activity against LDL oxidation induced by Cu^2+^, with an IC_50_ value of 5.66 ± 0.05 µg/mL, while the EtOAc fraction of *E. stolonifera* showed very high inhibition, with an IC_50_ value of 3.04 ± 0.13 µg/mL. Among the isolated phlorotannins (phloroglucinol, dioxinodehydroeckol, eckol, phlorofucofuroeckol -A, dieckol, and 7-phloroeckol), phlorofucofuroeckol-A and dieckol were found to be potent Cu^2+^-induced LDL oxidation inhibitors. Eckol showed moderate inhibition exhibiting an IC_50_ value of 7.47 µM. Because the production of conjugated dienes indicates the start of LDL oxidation, the lag time can be used as a measure of LDL oxidation resistance. Eckol extended the lag time to 54 min at 1 µM, 97 min at 5 µM, and 117 min at 10 µM. Probucol, the positive control, increased the lag time to 55 min at 1 µM, 74 min at 5 µM, and 91 min at 10 µM, thus, eckol was more effective than probucol at inhibiting conjugated diene formation [81].

### 2.15. Anti-Tyrosinase Activity

Tyrosinase is a copper-containing enzyme that catalyzes the production of melanin and other pigments from l-tyrosine by oxidation, as in the blackening of a peeled or sliced potato exposed to air [146]. Improvement of hyperpigmentation related disorders such as melasma and age spots is related to a decrease in tyrosinase activity [147]. Out of the seventeen tested seaweeds, only *E. stolonifera* showed high tyrosinase inhibitory activity. The phlorotannins isolated from the active EtOAc-soluble fraction of *E. stolonifera* were phloroglucinol, dioxinodehydroeckol, eckol, phlorofucofuroeckol-A, and dieckol showing IC_50_ values of 92.8, 126, 33.2, 177, and 2.16 µg/mL, respectively, against mushroom tyrosinase. Phloroglucinol and eckol displayed higher inhibition than arbutin, and dieckol showed a three times higher inhibition than kojic acid. A non-competitive inhibition against mushroom tyrosinase was exhibited by eckol, phlorofucofuroeckol-A and dieckol with *K_i_* values of 1.9 × 10^−5^, 1.4 × 10^−3^ and 1.5 × 10^−5^ M, respectively [82]. 

In a similar study of tyrosinase inhibition, eckol derived from *E. cava* was evaluated on melanogenesis and its binding capacity using a molecular docking simulation. The impact of eckol on melanin synthesis was evaluated in B16F10 melanoma cells induced by the α-melanocyte stimulating hormone. Eckol (25–100 μM) suppressed melanin synthesis and tyrosinase activity by inhibiting tyrosinase, both TRP1 and TRP2, expression in α-MSH-stimulated B16F10 cells. As compared to 50–100 μM eckol, 350 µM of arbutin (positive control) presented a weaker inhibition on tyrosinase activity [148].

### 2.16. Miscellaneous

As in other fields, eckol has shown promising results in anti-hair loss, anti-hearing loss, and anti-osteoporosis treatments.

Hair loss, also known as alopecia or baldness, describes the loss of hair from the head or body, with severity varying from a small section to the whole body. One study evaluated the effect of *E. cava* on the promotion of hair growth. Treatment with *E. cava* enzymatic extract and compounds isolated from it resulted in the proliferation of immortalized vibrissa dermal papilla cells (DPCs). The DPCs were treated with compounds isolated from *E. cava* enzymatic extract including eckol, dieckol, phloroglucinol, or triphlorethol-A at a concentration of 0.005, 0.01, 0.05, 0.1, 0.5, 1, and 10 µg/mL for 4 days, which resulted in proliferation of DPCs by 100.8%, 106.1%, 120.3%, 108.5%, 107.8%, 105.4%, and 104.1%, respectively. Since the mitotic effect on NIH3T3 fibroblasts via the K_ATP_ channel opening leads to the promotion of hair growth, the *E. cava* enzymatic extract, eckol, dieckol, phloroglucinol, and triphlorethol-A were tested for their ability on hair growth. Eckol increased the proliferation of NIH3T3 fibroblasts to some extent as compared with the control group. Eckol and dieckol also inhibited 5α-reductase activity in a dose-dependent manner which is important for preventing hair loss [149].

In another study, the protective effect of eckol was linked to anti-hearing loss activity by preventing the temporary threshold shift (TTS). The implications of noise-induced hearing loss (NIHL) has received a lot of consideration among teenagers in recent years. Temporary threshold shift, which is a type of NIHL, accelerates the age-related hearing loss even if it is a transient one. As the primary cause of TTS is ROS, the protective activity of polyphenolic extract of *E. cava* (PPEE) was evaluated which resulted in decreased ROS levels. The notable compounds in PPEE were typical polyphenols found in *E. cava* which included dieckol (16.8%), phlorofucofuroeckol-A (3.5%), and eckol (1.9%), contributing to its anti-oxidant potential. The intraperitoneal administration of PPEE (100 mg/kg) and saline to the mice with exposure to noise significantly decreased the auditory brainstem response threshold shifts and provided a significant degree of protection in relation to the distortion product otoacoustic emission levels. Thus, eckol containing PPEE may have good potential to break the vicious cycle of TTS [150].

The bone-related disorder leading to an imbalance in bone mass is osteoporosis treated by the formation of bone tissue through osteoblast differentiation together with suppression of the possible causes of bone volume loss [151]. But the close connection between the onset of osteoblastogenesis and obesity and DM are major concerns for treatment in the aged patients [152,153]. Since natural products are in trend to attenuate to osteoporosis, *E. cava* and its bioactive constituents were tested for their effects on the adipogenic differentiation of 3T3-L1 fibroblasts and the osteoblast differentiation of MC3T3-E1 pre-osteoblasts. Triphlorethol-A, eckol, and dieckol at 20 µM exhibited anti-adipogenic activity by reducing lipid aggregation and by suppressing the expression of adipogenic differentiation markers. They also enhanced osteoblast differentiation which was shown by raised alkaline phosphatase action, increased levels of indicators related to osteoblastogenesis, and also affected intracellular calcification. Dieckol was the most active compound followed by triphlorethol-A and eckol against osteoporotic conditions [154].

## 3. Discussion

As outlined in the above review, eckol has been evaluated copiously for a broad spectrum of activities to establish it as a potential therapeutic agent. According to various experimental results, eckol possesses a wide array of therapeutic properties. Through various studies [42,43], eckol had been established as an antioxidant; however, the evidence of its usefulness in cell-based experiments is limited. Hence, the results of the cell-based experiments could act as future references for in vivo experiments to doubtlessly highlight antioxidant potential. Eckol has also shown promising results in diabetes suppression. Since diabetes is incurable and can only be suppressed over time, scientists are always on the verge of finding new compounds and pathways to cure this disease. As eckol has been evaluated for its role in the existing signaling pathways against diabetes, it would be interesting to see the effect of eckol on the newly discovered pathways other than the evaluated ones. Furthermore, eckol had contraindications for not being useful in hepatoprotection at the cellular level [92], while it showed hepatoprotective activity in an in vivo model [48]. The possible reason for this discrepancy could be different toxicity-inducing agents employed in those studies, where eckol was ineffective against tacrine-induced toxicity in human liver cell line but exhibited a protective effect against CCl_4_-induced toxicity in mice model. Hence, why eckol failed to protect the liver cell against tacrine-induced toxicity needs further detailed research to know the exact mechanism involved. In neuroprotection, the GABA_A_–BZD receptor and dopamine receptors hold a good future for eckol due to its potent activity against these receptors [55,56]. The high activity of eckol in AChE enzyme inhibition but not in BChE inhibition [26] explains its selective nature, including moderate *h*MAO-B inhibition [53]. Although these enzyme inhibition effects displays its neuroprotective nature, cell-based and in vivo experiments are imperative to show its actual therpeutics. In several diseases requiring radiation therapy, including cancer, radioprotection is of the utmost importance. Although the in vivo experiments showed eckol as a radioprotector [62,63,64], there are still many complications and considerations while using it for human clinical trials, the correct dose of eckol required against the radiation being the utmost important part. Hence, these studies could be the references through which eckol could be further developed as a pharmaceutical product. Eckol showed a moderate protective role in skin cells against UV radiation [58,60] by scavenging ROS and reducing intracellular oxidative damage induced by UVB radiation. Further, the in vivo experiment [59] also supported eckol as a UV protector against skin carcinogenesis. Hence, eckol could be a potent UV protector in pharmaceuticals or cosmeceuticals. Regarding its anticoagulative property [16,120], the dibenzo-1,4-dioxane skeleton (dibenzo-*p*-dioxin-I,3,6,8-tetraol) of eckol seems to be one of the major factors involved in the anticoagulative effect which requires further investigation. Although eckol showed potent anticoagulative effect via thrombin activity inhibition, the activity was reduced in whole plasma [16] most probably due to the plasma–protein binding affecting its efficacy. Hence, searching for alternatives to increase its bioavailability could be good future research. Similarly, the antithrombotic activity of eckol [77] also supports the possibility of developing eckol as an anticoagulant. The antihypertensive effect of eckol was attributed to ACE-inhibition, and the closed-ring dibenzo-1,4-dioxane was crucial for the inhibition [28]. Still in vivo study is a requisite for establishing eckol as a pharmaceutical product. Furthermore, following the success in characterizing eckol as an anticancer agent via in vitro cell-based studies [75,76], eckol could further be tested in vivo to establish its full potential against this life-threatening disease. Several contraindications of eckol being less active [19,73] or inactive [74] against the allergic symptoms are reported. The compounds (fucodiphloroethol G, phlorofucofuroeckol A, dieckol, 8,8′-bieckol) other than eckol showed more potent activity, and all of them have the basic skeleton of eckol which could have a crucial role for antiallergic activity. So it is worth confirming the antiallergic property of eckol in vivo. In terms of the antiviral and antibacterial activity, eckol showed eminent results [29,67,124], but the production of proinflammatory cytokines in response to the antiviral activity of extract containing eckol was a contraindicatory point [67]. Even though the increase in pro-inflammatory cytokines may lead to inflammation, the subsequent basal expression levels of these cytokines after a significant increase in the early stage of an oral administration of extract indicate the immunomodulatory activity of the extract. Hence, the extract activated the anti-inflammatory response or suppressed the pro-inflammatory cytokines to control the extent of inflammatory condition. Eckol exhibiting a potent anti-inflammatory activity is a point to be noted, but the introduction of the dimers and isomers of eckol have surpassed the activity of eckol and have appeared to be more potent. Even though they are potent, the geometry of the aromatic ring and main configuration of the eckol skeleton is always critical for the suppressive property. The cell-based experiments related to tyrosinase inhibition by eckol could also be beneficial as future references for experiments on the living subjects. The potent anti-hyperlipidemic activity of eckol compared to probucol [81] and the suppression of high lipid levels in serum of hyperlipidemic rats [80] could be evidence to develop it as a pharmaceutical agent against hyperlipidemia. The anti-hair loss [149] and anti-hearing loss [150] activities of eckol show that it still holds potential in several unidentified fields other than ones that are stated in this review. Hence, a continuous evaluation of eckol in the future is important to increase its spectrum of activity.

## 4. Conclusions and Future Perspectives

Numerous efforts to establish natural biomarkers for the treatment of various ailments have improved our understanding of their functions and activity. Considering the diversity of natural biomarkers such as eckol, a definite push is required to further decode these kinds of compounds for their proper use. Eckol exhibiting several biological activities is extraordinary, particularly the involvement of eckol as an anti-oxidant, anti-diabetic, radioprotective, hepatoprotective, and anti-inflammatory agent to name some. The use of eckol as anti-hair loss and anti-hearing loss is a refreshing new discovery. Increasing the scope of eckol as a neuroprotective agent by evaluating it against G protein coupled dopamine receptors is also a novel exploration for the treatment of major neurodegenerative disorders like Alzheimer’s and Parkinson’s disease. Thus, introducing eckol to various new receptors related to different diseases, other than the usual ones, could be of great interest. Since eckol exhibits activity against life-threatening disease like cancer, further research in this particular field also holds great potential for eckol to be established as an anti-cancer agent. The major reason for the broad spectrum of activity related to eckol is possibly due to its structure. Even the other potent phlorotannins of the same family have a basic backbone of eckol. So, developing eckol as a single pharmaceutical product could be beneficial in the field of medicine. Hence, natural product-derived compounds hold a multitude of opportunities for the discovery of novel therapeutic agents. The shift in focus from synthetically developed agents to natural agents could be a stepping stone for further advancements in the treatment of various infirmities. In conclusion, these kinds of naturally derived drugs have immense potential for advancement in the pharmaceutical and medical fields, leading to a definite change in views.

## Figures and Tables

**Table 1:** Occurrence of eckol.

Scientific Name	Types of Algae	Extracts	References
*Ecklonia cava*	Brown algae	Methanol/Ethanol	[11,12]
*Ecklonia stolonifera*	Brown algae	Methanol/Ethanol	[13,14]
*Ecklonia kurome*	Brown algae	Methanol/Ethanol	[15,16]
*Ecklonia maxima*	Brown algae	Methanol	[17]
*Eisenia bicyclis*	Brown algae	Methanol	[18]
*Eisenia arborea*	Brown algae	Methanol:Chloroform	[19]
*Myagropsis myagroides*	Brown algae	Ethanol	[20]

**Table 2:** Summary of the biological activities of eckol.

Pharmacological Effect	Experimental Methods	Pathway/Mode	Application	References
Antioxidant	Radical 2,2-diphenyl-1-picrylhydrazyl (DPPH), hydroxyl, peroxyl, and superoxide anion scavenging activity using the electron spin resonance spectrometry (ESR) technique. Cellular reactive oxygen species (ROS) determination using Dichloro-dihydro-fluorescein diacetate (DCFH-DA). Measurement of cellular glutathione (GSH) level. Myeloperoxidase (MPO) assay.	Showed significant radical scavenging activity. Significantly reduced ROS level at 50 μM concentration. Showed moderate inhibitory activity against membrane protein oxidation. Moderately enhanced cellular GSH level. Inhibited MPO activity moderately, inhibited ROS generation in kidney homogenate	In vitro	[41]
	Heme oxygenase (HO)-1 activity/expression level via immunoprecipitation and Western blotting.	Regulated HO-1 via Nrf2 nuclear translocation and extracellular related kinase (Erk), Phosphoinositide 3-kinase (PI3K)/protein kinase B (PKB, also known as Akt) modulation.	In vitro	[42]
	DPPH radical scavenging assay, measurement of hydrogen peroxide, catalase activity, and lipid peroxidation assay. Western blotting, electrophoretic mobility shift assay (EMSA), and transient transfection and NF-κB luciferase assay.	Exhibited significant scavenging of DPPH radical, hydrogen peroxide (H_2_O_2_), hydroxy radical, intracellular ROS, prevention of lipid peroxidation. Phosphorylated extracellular signal-regulated kinase and activity of NF-κB.	In vitro	[43]
	Measurement of ROS and Ca^2+^ levels using dihydrorhodamine (DHR) 123 and Rhod-2 acetoxymethyl-ester (AM) dye. Mitochondrial succinate dehydrogenase activity. Western blot analysis, reverse transcriptase polymerase chain reaction (RT-PCR), and measurement of manganese superoxide dismutase (MnSOD) activity.	Decreased H_2_O_2_-induced mitochondrial ROS levels and attenuated ROS-generated mitochondrial Ca^2+^ levels. Prevented dysfunction of mitochondria following ROS and Ca^2+^ induction. Induction of MnSOD following activation of AMP-activated protein kinase/forkhead box O3a (AMPK/FoxO3a).	In vitro	[44]
	Radical scavenging activity using dichloro-dihydro-fluorescein (DCF) method, lipid peroxidation assay, and image analysis. Measurement of intracellular Ca^2+^ level using Fluo-3 AM in HT22 cells.	Scavenged intracellular ROS and repressed accumulation of ROS, prevented of lipid peroxidation, inhibited H_2_O_2_-induced cell death, and Ca^2+^ release in HT22 cells.	In vitro	[11]
	Radical scavenging activity using DCF method, lipid peroxidation assay, and image analysis. Cell death evaluation using acridine orange.	Scavenged intracellular ROS, prevented of lipid peroxidation. Decreased cell death in zebrafish embryos induced by2,2’-Azobis(2-amidinopropane) dihydrochloride (AAPH).	In vitro	[21]
	Cellular lipid peroxidation assay qRT-PCR analysis, enzyme-linked immunosorbent assay (ELISA).	Attenuated cellular lipid peroxidation, expression level of inflammatory cytokines (tumor necrosis factor (TNF)-α, interleukin (IL)-1β, IL-6, and IL-8).	In vitro	[12]
	Weight gain test using methyl α-linolenate.	Showed weight gain indicating its antioxidant potential.	In vitro	[18]
	Hydrophilic oxygen radical absorbance capacity (H-ORAC) assay.	Scavenged peroxyl radicals induced by AAPH.	In vitro	[45]
Anti-diabetic	Protein tyrosine phosphatase (PTP1B) and α-glucosidase inhibitory assay.	Inhibited α-glucosidase enzyme activity indicating its potential to delay the postprandial increase of blood glucose level.	In vitro	[17]
	Advanced glycation end products (AGEs), α-amylase, rat lens aldose reductase (RLAR) inhibitory assay.	Inhibited AGEs formation, α-amylase, rat lens aldose reductase activity.	In vitro	[46]
	Spectrophotometric assay on carbohydrate-hydrolyzing enzymes. Starch and sucrose tolerance test.	Inhibited α-amylase and α-glucosidase enzyme activity. Enhanced glucose tolerance and reduced fasting blood glucose, insulin, fructosamine, and glycoalbumin level.	In vitro/ In vivo	[15]
Hepatoprotective	2-(4-Iodophenyl)-3-(4-nitrophenyl)-5-(2,4-disulfophenyl)-2 tetrazolium (WST-1) colorimetric assay.	Scavenged free radicals; inhibited trichloromethyl free radicals (CCl_3_·), and trichloromethyl peroxy radicals (CCl_3_OO·) through cytochrome P450 systems-related CCl_4_ metabolism	In vitro	[25]
	Tacrine-induced cytotoxicity assay.	Altered intracellular glutathione concentrations leading to inhibition of ROS generation and lipid peroxidation.	In vitro	[47]
	Western immunoblot, confocal microscopic analysis.	Diminished the expression of Fas-mediated cell-death proteins (tBid, caspase-3, and poly (ADP-ribose) polymerase). Decreased the release of cytochrome c from mitochondria to cytosol.	In vitro	[14]
	Enzymatic colorimetric method. Terminal deoxynucleotidyl transferase-mediated dUTP nick end labelling (TUNEL) staining. ELISA. Bicinchoninic acid assay (BCA).	Increased alanine transaminase (ALT) and aspartate aminotransferase (AST) levels in serum and ameliorated morphological liver injury in rat. Protected rat hepatocytes from CCl_4_-mediated apoptosis. Reduced malondialdehyde (MDA) formations, TNF-α, in IL-1β, IL-6 and IL-10. Enhanced superoxide dismutase (SOD), glutathione peroxidase (GSH-Px) activities. Increased GSH content.	In vivo	[48]
	Online HPLC- 2,2’-azino-bis (3-ethylbenzothiazoline-6-sulphonic acid (ABTS) radical scavenging assay.	Scavenged ABTS radical and decreased oxidative stress in *t*-BHP induced cell death in HepG2 cells.	In vitro	[49]
	3-(4,5-dimethylthiazol-2-yl)-2,5-diphenyltetrazolium bromide (MTT) assay. Nuclear staining with Hoechst 33342.	Reduced the formation of apoptotic bodies in ethanol induced damage in Chang liver cells	In vitro	[50]
Neuroprotective	β-secretase 1 (BACE1) inhibition assay.	Potently inhibited BACE1 activity	In vitro	[51]
	Cholinesterase enzyme inhibition assay.	Inhibited acetyl and butyl cholinesterase enzyme activity.	In vitro	[52]
	Monoamine oxidase (MAO) enzyme inhibition assay.	Exhibited good inhibition in MAOs-A and-B enzyme activity.	In vitro	[53]
	MTT assay in PC12 cells. Determination of ROS level in Aβ-treated PC12 cells.	Protected PC12 cells against Aβ-induced cytotoxicity. Inhibited intracellular ROS and Ca^2+^ generation.	In vitro	[54]
	GABA_A_–Benzodiazepine (BZD) receptor-binding assay and pentobarbital-induced sleep test.	Showed allosteric modulation of the GABA_A_–BZD receptor.	In vitro/ In vivo	[55]
	G-protein coupled receptors (GPCRs) cell based functional assays.	Exhibited dual human dopamine D_3_/D_4_ agonist activity.	In vitro	[56]
Anti-photo aging	Luciferase reporter gene assay, Western blot analysis and RT-PCR.	Inhibition on NF-κB and AP-1 reporter activity. Inhibited the expression of matrix metalloproteinase 1 (MMP-1) in TNF-α-induced human dermal fibroblasts and collagen degradation by MMP-1.	In vitro	[57]
	ROS level determination using DCF method in ultraviolet (UV) B radiated HaCaT cells, Lipid peroxidation assay, single-cell gel electrophoresis (Comet Assay), and protein carbonyl formation. Mitochondrial membrane potential (Δψm) analysis.	Decreased UVB-induced intracellular ROS level. Inhibited membrane lipid peroxidation, protein carbonylation and cellular DNA damage. Protected against UVB-induced apoptosis.	In vitro	[58]
	Real-time RT-PCR analysis, Western blot analysis, prostaglandin (PG) E_2_ enzyme immunoassay, immunohistochemical detection of proliferating nuclear cell antigen.	Suppressed the expression of cyclooxygenase-2 (COX-2) leading to decrease in COX-2 protein production in UVB-induced skin carcinogenesis. Suppressed the gene expression of PGE_2_ in UVB-induced skin carcinogenesis. Significantly decreased the proliferation percentage of the cells in the epidermis	In vitro	[59]
	DCFH-DA and MTT assay, Comet assays.	Reduction in intracellular ROS accumulation, Reduction in fragmentation and destruction of irradiated cells.	In vitro	[60]
	Measurement of ROS level in UVB induced photo damage in zebrafish embryos. Detection of nitric oxide using diaminofluorophore 4-amino-5-methylamino2′,7′difluorofluorescein diacetate (DAF-FM DA).	Reduced reactive oxygen species and nitric oxide levels.	In vivo	[61]
Radio protective	Hematoxylin and eosin staining and immunohistochemistry of jejunal crypt cells. Western blot analysis.	Protected the viability of lymphocytes and intestinal cells from radiation induced apoptosis. Decreased pro-apoptotic p53 and Bax and increased antiapoptotic B-cell lymphoma-2 (Bcl-2) concentration.	In vivo/ In vitro	[62]
	Jejunal crypt assay. Apoptosis assay (hematoxylin-eosin and an in situ DNA end- labeling (ISEL) technique).	Enhanced the jejunal crypt survival. Protection against apoptosis induced by radiation in the mice jejunal crypts.	In vivo	[63]
	Colony-forming units (CFU) assay, alkaline comet assay, 3H-thymidine, incorporation assay, flow cytometry.	Enhances hematopoietic recovery. Reduced DNA damage in lymphocytes. Induced splenocyte proliferation. Increased the populations of T and B cells.	In vivo	[64]
	Intracellular reactive oxygen species measurement, single cell gel electrophoresis (Comet assay), 8-hydroxy-2′-deoxyguanosine (8-OHdG) assay and lipid peroxidation assay, mitochondrial membrane potential (Δψ) analysis electrophoretic mobility shift assay transient transfection and activator protein (AP)-1 luciferase assay.	Scavenging effect on reactive oxygen species, inhibition of damage of cellular DNA and peroxidation of membrane lipid in γ-ray irradiated cells. Protected against apoptosis by increasing Bcl-2 expression and decreasing Bax expression in γ-ray irradiated cells. Suppression of the SEK1-JNK-AP-1 pathway induced by γ-ray irradiation.	In vitro	[65]
Anti-bacterial and Anti-viral	Disk-diffusion assay, time–kill assay, checkerboard dilution test, antibacterial activity assay.	Antibacterial activity against methicillin-resistant *Staphylococcus aureus*, *Salmonella* species and food-borne pathogenic bacteria with additive effect of eckol and ampicillin. Reduction of CFU.	In vitro	[29]
	Chemiluminescent neuraminidase inhibition assay. Recombinant influenza virus neuraminidase inhibition assay.	Inhibition of influenza virus neuraminidase binding to free enzyme and/or product-bound enzyme.	In vitro	[66]
	Plaque reduction assay (Co-, pre- and post-exposure antiviral activity), qRT-PCR.	Inhibitory activity against viral hemorrhagic septicemia virus (VHSV) in the fathead minnow (FHM) cell line. Significantly induced inflammatory cytokine responses (interleukin (IL)-1β, IL-6, and IFN-γ) and interferon (IFN)-α/β, interferon stimulated gene (ISG) 15 and Mx were significantly activated.	In vitro/ In vivo	[67]
	Severe acute respiratory syndrome coronavirus 3C-like proteinase (SARS-CoV 3CL^pro^) cis-cleavage assay.	Inhibition through SARS-CoV 3CL^pro^ trans/cis-cleavage.	In vitro	[68]
Anti-inflammatory	The flux of albumin in a dual chamber system monitored in lipopolysaccharide (LPS)-mediated human umbilical vein endothelial cells (HUVECs) and in mice. Acetic acid-induced vascular permeability in mice.	Inhibited LPS-mediated barrier disruption and trans endothelial migration of leukocytes to human endothelial cells. Suppressed acetic acid induced-hyper permeability and carboxymethylcellulose-induced leukocytes migration in vivo.	In vitro/ In vivo	[69]
	Competitive ELISA for high mobility group box 1 (HMGB1), permeability assay in vitro and in vivo, cell–cell adhesion assay, migration assay in vitro and in vivo.	Inhibited HMGB1 release and HMGB1 induced barrier disruption. Suppressed the expressions of cell adhesion molecule (CAM) (VCAM-1, ICAM-1 and E-selectin), inhibited the binding of monocytes to HMGB1-stimulated endothelial cells and inhibited trans-endothelial migration (TEM).	In vitro/ In vivo	[24]
	Assays of mouse ear edema induced by arachidonate (AA), 12-O-tetradecanoylphorbol-13-acetate (TPA), and oxazolone (OXA). Cell stimulation, anti-degranulation assay and qRT-PCR	Suppressed mouse ear swelling and the release of histamine, leukotriene B_4_, prostaglandin E_2_, and mRNA expression, and/or the activity of COX-2 in rat basophilic leukemia (RBL)-2H3 cells, inhibited delayed-type (type IV) allergic reactions induced by oxazolone	In vivo	[70]
	RNA extraction, RT-PCR, Western blot analysis	Inhibited the expression and production of proinflammatory mediators and cytokines in HaCat cells. Inhibited phosphorylation of Akt and activation of NF-κb mediated by *Propionibacterium acnes*.	In vitro	[71]
	Secretory phospholipase A_2_s, soybean lipoxygenase (LOX), 5-lipoxygenase, and COX-1 and COX-2 inhibitory assay.	Inhibition on secretory phospholipase A_2_s, LOX and COX activity.	In vitro	[72]
Anti-histamine	Histamine release assay. Rat basophilic leukemia (RBL)-2H3 cell stimulation and assay for β-hexosaminidase release. Flow cytometry analysis.	Inhibition on the degranulation of KU812 cells stimulated by immunoglobulin E (IgE) and anti-IgE antibodies. Inhibition on the degranulation of RBL-2H3 cells mediated by IgE stimulation via a granular enzyme (β-hexosaminidase). Inhibited binding of IgE and FcεRI receptor in KU812 cells.	In vitro	[73]
	Assay of hyaluronidase.	Showed inhibition in hyaluronidase activity.	In vitro	[74]
Anti-hypertensive	Angiotensin converting enzyme (ACE) inhibitory activity assay. Determination of nitric oxide (NO) production.	ACE inhibitory and peroxynitrite scavenging properties.	In vitro	[28]
Anti-cancer	Flow cytometric analysis, soft agar colony formation assay, and invasion assay. PI3K kinase assay, Raf-1 kinase assay, and activated Ras affinity precipitation assay.	Suppressed expression of the glioma stem-like cell markers and the self-renewal-related proteins without cell death. Inhibited PI3K-Akt and Ras-Raf-1-Erk signaling pathways.	In vitro	[75]
	MTT reduction assay on a limited variety of cancer cell lines.	Exhibited anti-proliferation effect.	In vitro	[76]
Anti-coagulant	Anti-α_2_ plasmin, anti-α_2_ macroglobulin, anti-α_1_ anti thrombin (AT), anti-ATIII, anti-ATIII-heparin complex, anti-CI-inactivator activity assays and assay of inhibition of proteases. Evaluation on effects of fibrinolysis in human plasma induced by urokinase.	Potentiation of thrombolytic activity. Enhanced the effects fibrinolysis in human plasma induced by urokinase.	In vitro	[16]
	Anticoagulation assay and thrombin activity assay and Factor Xa (FXa) activity assay. ELISA for plasminogen activator inhibitor (PAI)-1 and tissue plasminogen activator (t-PA).	Prolonged activated partial thromboplastin time (aPTT) and prothrombin time (PT) significantly and inhibited the generation of thrombin or FXa in human umbilical vein endothelial cells (HUVECs). Inhibited TNF-α induced PAI-1 production and the ratio between PAI-1 and t-PA (procoagulant and hypofibrinolysis activities).	In vitro/ In vivo	[77]
Anti-obesity	Measurement of fat mass. Plasma analysis and intraperitoneal glucose tolerance test. Analysis of mRNA by quantitative real-time PCR. Quantification of liver triglyceride (TG) content.	Reduction of body weight, adiposity, ALT, and cholesterol Reduction of blood glucose levels. Increased mRNA expression of adipogenic ttyt 777 genes, and mRNA expression of inflammatory cytokines. Decreased macrophage marker gene. Inhibited intrahepatic lipid accumulation and hepatic lipogenic gene mRNA expression.	In vivo	[78]
	Glucose utilization assay and triglyceride accumulation assay. Western blot analysis.	Suppressed glucose utilization, TG build-up, and downregulation of CCAAT-enhancer-binding proteins (C/EBP)α in 3T3-L1 adipocytes.	In vitro	[27]
	Assay of pancreatic lipase activity.	Inhibited pancreatic lipase activity.	In vitro	[79]
Anti-hyperlipidemic	Enzymatic colorimetric methods for biochemical estimations by using commercial kits.	Reduced the level of TG, total cholesterol (TC), and low-density lipoprotein-cholesterol (LDL-C) and increased level of the high-density lipoprotein-cholesterol (HDLC).	In vivo	[80]
	Thiobarbituric acid reactive substances (TBARS) assay.	Inhibitory activity against Cu^2+^-induced LDL oxidation and conjugated diene formation (TBARS assay).	In vitro	[81]
Anti-tyrosinase	Measurement of cellular melanin contents and tyrosinase activity in B16F10 melanoma cells. Western blot analysis.	Suppressed tyrosinase activity and melanin synthesis. Decreased the expression of cellular tyrosinase enzyme, tyrosinase-related protein (TRP) 1, and TRP2.	In vitro	[82]

## References

[1] Muhammad S.A., Muhammad J., Muhammad S., Muhammad K.P., Shaista H., Viqar U.A. (2000). Metabolites of marine algae collected from Karachi-coasts of Arabian Sea. Nat. Prod. Sci..

[2] Chen J.H., Lim J.D., Sohn E.H., Choi Y.S., Han E.T. (2009). Growth-inhibitory effect of a fucoidan from brown seaweed *Undaria pinnatifida* on Plasmodium parasites. Parasitol. Res..

[3] Cox S., Abu-Ghannam N., Gupta S. (2010). An assessment of the antioxidant and antimicrobial activity of six species of edible Irish seaweeds. Int. Food Res. J..

[4] Rupérez P., Ahrazem O., Leal J.A. (2002). Potential antioxidant capacity of sulfated polysaccharides from the edible marine brown seaweed *Fucus vesiculosus*. J. Agric. Food Chem..

[5] Taskin E., Ozturk M., Kurt O. (2007). Antibacterial activities of some marine algae from the Aegean Sea (Turkey). Afr. J. Biotechnol..

[6] Dhargalkar V., Pereira N. (2005). Seaweed: Promising plant of the millennium. Sci. Cult..

[7] Zou Y., Qian Z.J., Li Y., Kim M.M., Lee S.H., Kim S.K. (2008). Antioxidant effects of phlorotannins isolated from *Ishige okamurae* in free radical mediated oxidative systems. J. Agric. Food Chem..

[8] Airanthi M.W.A., Hosokawa M., Miyashita K. (2011). Comparative antioxidant activity of edible Japanese brown seaweeds. J. Food Sci..

[9] Thomas N.V., Kim S.K. (2011). Potential pharmacological applications of polyphenolic derivatives from marine brown algae. Environ. Toxicol. Pharmacol..

[10] Singh I.P., Bharate S.B. (2006). Phloroglucinol compounds of natural origin. Nat. Prod. Rep..

[11] Kang S.M., Cha S.H., Ko J.Y., Kang M.C., Kim D., Heo S.J., Kim J.S., Heu M.S., Kim Y.T., Jung W.K. (2012). Neuroprotective effects of phlorotannins isolated from a brown alga, *Ecklonia cava*, against H_2_O_2_-induced oxidative stress in murine hippocampal HT22 cells. Environ. Toxicol. Pharmacol..

[12] Lee J.W., Seok J.K., Boo Y.C. (2018). Ecklonia cava extract and dieckol attenuate cellular lipid peroxidation in keratinocytes exposed to PM10. Evid. Based Complement. Alternat. Med..

[13] Kang H.S., Chung H.Y., Kim J.Y., Son B.W., Jung H.A., Choi J.S. (2004). Inhibitory phlorotannins from the edible brown alga *Ecklonia stolonifera* on total reactive oxygen species (ROS) generation. Arch. Pharm. Res..

[14] Lee M.S., Shin T., Utsuki T., Choi J.S., Byun D.S., Kim H.R. (2012). Isolation and identification of phlorotannins from *Ecklonia stolonifera* with antioxidant and hepatoprotective properties in tacrine-treated HepG2 cells. J. Agric. Food Chem..

[15] Xu H.L., Kitajima C., Ito H., Miyazaki T., Baba M., Okuyama T., Okada Y. (2012). Antidiabetic effect of polyphenols from brown alga *Ecklonia kurome* in genetically diabetic KK-Ay mice. Pharm. Biol..

[16] Nakayama Y., Takahashi M., Fukuyama Y., Kinzyo Z. (1989). An anti-plasmin inhibitor, eckol, isolated from the brown alga *Ecklonia kurome* Okamura. Agric. Biol. Chem..

[17] Rengasamy K.R., Aderogba M.A., Amoo S.O., Stirk W.A., Van Staden J. (2013). Potential antiradical and alpha-glucosidase inhibitors from *Ecklonia maxima* (Osbeck) Papenfuss. Food Chem..

[18] Nakamura T., Nagayama K., Uchida K., Tanaka R. (1996). Antioxidant activity of phlorotannins isolated from the brown alga *Eisenia bicyclis*. Fish. Sci..

[19] Sugiura Y., Matsuda K., Yamada Y., Nishikawa M., Shioya K., Katsuzaki H., Imai K., Amano H. (2007). Anti-allergic phlorotannins from the edible brown alga, *Eisenia arborea*. Food Sci. Technol. Res..

[20] Joung E.J., Lee M.S., Choi J.W., Kim J.S., Shin T., Jung B.M., Yoon N.Y., Lim C.W., Kim J.I., Kim H.R. (2012). Anti-inflammatory effect of ethanolic extract from *Myagropsis myagroides* on murine macrophages and mouse ear edema. BMC Complement. Altern. Med..

[21] Kang M.C., Cha S.H., Wijesinghe W., Kang S.M., Lee S.H., Kim E.A., Song C.B., Jeon Y.J. (2013). Protective effect of marine algae phlorotannins against AAPH-induced oxidative stress in zebrafish embryo. Food Chem..

[22] Jun Y.J., Lee M., Shin T., Yoon N., Kim J.H., Kim H.R. (2014). Eckol enhances heme oxygenase-1 expression through activation of Nrf2/JNK pathway in HepG2 cells. Molecules.

[23] Sugiura Y., Tanaka R., Katsuzaki H., Imai K., Matsushita T. (2013). The anti-inflammatory effects of phlorotannins from *Eisenia arborea* on mouse ear edema by inflammatory inducers. J. Funct. Foods.

[24] Kim T.H., Ku S.K., Lee T., Bae J.S. (2012). Vascular barrier protective effects of phlorotannins on HMGB1-mediated proinflammatory responses in vitro and in vivo. Food Chem. Toxicol..

[25] Jung H.A., Kim J.I., Choung S.Y., Choi J.S. (2014). Protective effect of the edible brown alga *Ecklonia stolonifera* on doxorubicin-induced hepatotoxicity in primary rat hepatocytes. J. Pharm. Pharmacol..

[26] Kannan R.R., Aderogba M.A., Ndhlala A.R., Stirk W.A., Van Staden J. (2013). Acetylcholinesterase inhibitory activity of phlorotannins isolated from the brown alga, *Ecklonia maxima* (Osbeck) Papenfuss. Food Res. Int..

[27] Kim I.H., Nam T.J. (2017). Enzyme-treated *Ecklonia cava* extract inhibits adipogenesis through the downregulation of C/EBPα in 3T3-L1 adipocytes. Int. J. Mol. Med..

[28] Jung H.A., Hyun S.K., Kim H.R., Choi J.S. (2006). Angiotensin-converting enzyme I inhibitory activity of phlorotannins from *Ecklonia stolonifera*. Fish. Sci..

[29] Choi J.G., Kang O.H., Brice O.O., Lee Y.S., Chae H.S., Oh Y.C., Sohn D.H., Park H., Choi H.G., Kim S.G. (2010). Antibacterial activity of *Ecklonia cava* against methicillin-resistant *Staphylococcus aureus* and *Salmonella* spp.. Foodborne Pathog. Dis..

[30] Pangestuti R., Kim S.K. (2011). Biological activities and health benefit effects of natural pigments derived from marine algae. J. Funct. Foods..

[31] Shanmugam M., Mody K. (2000). Heparinoid-active sulphated polysaccharides from marine algae as potential blood anticoagulant agents. Curr. Sci..

[32] Abdul Q.A., Choi R.J., Jung H.A., Choi J.S. (2016). Health benefit of fucosterol from marine algae: A review. J. Sci. Food Agric..

[33] Smit A.J. (2004). Medicinal and pharmaceutical uses of seaweed natural products: A review. J. Appl. Phycol..

[34] Koirala P., Jung H.A., Choi J.S. (2017). Recent advances in pharmacological research on Ecklonia species: A review. Arch. Pharm. Res..

[35] Yu K.X., Jantan I., Ahmad R., Wong C.L. (2014). The major bioactive components of seaweeds and their mosquitocidal potential. Parasitol. Res..

[36] Gupta S., Abu-Ghannam N. (2011). Recent developments in the application of seaweeds or seaweed extracts as a means for enhancing the safety and quality attributes of foods. Innov. Food Sci. Emerg. Technol..

[37] Wijesinghe W., Jeon Y.J. (2011). Biological activities and potential cosmeceutical applications of bioactive components from brown seaweeds: A review. Phytochem. Rev..

[38] Wijesinghe W., Jeon Y.J. (2012). Exploiting biological activities of brown seaweed *Ecklonia cava* for potential industrial applications: A review. Int. J. Food Sci. Nutr..

[39] Wijesekara I., Yoon N.Y., Kim S.K. (2010). Phlorotannins from *Ecklonia cava* (Phaeophyceae): Biological activities and potential health benefits. Biofactors.

[40] Shin T., Ahn M., Hyun J.W., Kim S.H., Moon C. (2014). Antioxidant marine algae phlorotannins and radioprotection: A review of experimental evidence. Acta Histochem..

[41] Li Y., Qian Z.J., Ryu B., Lee S.H., Kim M.M., Kim S.K. (2009). Chemical components and its antioxidant properties in vitro: An edible marine brown alga, *Ecklonia cava*. Bioorg. Med. Chem..

[42] Kim K.C., Kang K.A., Zhang R., Piao M.J., Kim G.Y., Kang M.Y., Lee S.J., Lee N.H., Surh Y.J., Hyun J.W. (2010). Up-regulation of Nrf2-mediated heme oxygenase-1 expression by eckol, a phlorotannin compound, through activation of Erk and PI3K/Akt. Int. J. Biochem. Cell Biol..

[43] Kang K.A., Lee K.H., Chae S., Zhang R., Jung M.S., Lee Y., Kim S.Y., Kim H.S., Joo H.G., Park J.W. (2005). Eckol isolated from *Ecklonia cava* attenuates oxidative stress induced cell damage in lung fibroblast cells. FEBS Lett..

[44] Kim A.D., Kang K.A., Piao M.J., Kim K.C., Zheng J., Yao C.W., Cha J.W., Hyun C.L., Kang H.K., Lee N.H. (2014). Cytoprotective effect of Eckol against oxidative stress-induced mitochondrial dysfunction: Involvement of the FoxO3a/AMPK pathway. J. Cell. Biochem..

[45] Fujii Y., Tanaka R., Miyake H., Tamaru Y., Ueda M., Shibata T. (2013). Evaluation for antioxidative properties of phlorotannins isolated from the brown alga *Eisenia bicyclis*, by the H-ORAC method. Food Nutr. Sci..

[46] Jung H.A., Yoon N.Y., Woo M.H., Choi J.S. (2008). Inhibitory activities of extracts from several kinds of seaweeds and phlorotannins from the brown alga *Ecklonia stolonifera* on glucose-mediated protein damage and rat lens aldose reductase. Fish. Sci..

[47] Kim Y.C., An R.B., Yoon N.Y., Nam T.J., Choi J.S. (2005). Hepatoprotective constituents of the edible brown alga *Ecklonia stolonifera* on tacrine-induced cytotoxicity in HepG2 cells. Arch. Pharm. Res..

[48] Li S., Liu J., Zhang M., Chen Y., Zhu T., Wang J. (2018). Protective effect of eckol against acute hepatic injury induced by carbon tetrachloride in mice. Mar. Drugs.

[49] Kim S.M., Kang K., Jeon J.S., Jho E.H., Kim C.Y., Nho C.W., Um B.H. (2011). Isolation of phlorotannins from *Eisenia bicyclis* and their hepatoprotective effect against oxidative stress induced by *tert*-butyl hyperoxide. Appl. Biochem. Biotechnol..

[50] Kang M.C., Kim K.N., Kang S.M., Yang X., Kim E.A., Song C.B., Nah J.W., Jang M.K., Lee J.S., Jung W.K. (2013). Protective effect of dieckol isolated from *Ecklonia cava* against ethanol caused damage in vitro and in zebrafish model. Environ. Toxicol. Pharmacol..

[51] Jung H.A., Oh S.H., Choi J.S. (2010). Molecular docking studies of phlorotannins from *Eisenia bicyclis* with BACE1 inhibitory activity. Bioorg. Med. Chem. Lett..

[52] Yoon N.Y., Chung H.Y., Kim H.R., Choi J.S. (2008). Acetyl-and butyrylcholinesterase inhibitory activities of sterols and phlorotannins from *Ecklonia stolonifera*. Fish. Sci..

[53] Jung H.A., Roy A., Jung J.H., Choi J.S. (2017). Evaluation of the inhibitory effects of eckol and dieckol isolated from edible brown alga *Eisenia bicyclis* on human monoamine oxidases A and B. Arch. Pharm. Res..

[54] Ahn B.R., Moon H.E., Kim H.R., Jung H.A., Choi J.S. (2012). Neuroprotective effect of edible brown alga *Eisenia bicyclis* on amyloid beta peptide-induced toxicity in PC12 cells. Arch. Pharm. Res..

[55] Cho S., Yang H., Jeon Y.J., Lee C.J., Jin Y.H., Baek N.I., Kim D., Kang S.M., Yoon M., Yong H. (2012). Phlorotannins of the edible brown seaweed *Ecklonia cava* Kjellman induce sleep via positive allosteric modulation of gamma-aminobutyric acid type A–benzodiazepine receptor: A novel neurological activity of seaweed polyphenols. Food Chem..

[56] Paudel P., Seong S.H., Wu S., Park S., Jung H.A., Choi J.S. (2019). Eckol as a potential therapeutic against neurodegenerative diseases targeting dopamine D3/D4 receptors. Mar. Drugs.

[57] Joe M.J., Kim S.N., Choi H.Y., Shin W.S., Park G.M., Kang D.W., Kim Y.K. (2006). The inhibitory effects of eckol and dieckol from *Ecklonia stolonifera* on the expression of matrix metalloproteinase-1 in human dermal fibroblasts. Biol. Pharm. Bull..

[58] Piao M.J., Lee N.H., Chae S., Hyun J.W. (2012). Eckol inhibits ultraviolet B-induced cell damage in human keratinocytes via a decrease in oxidative stress. Biol. Pharm. Bull..

[59] Hwang H., Chen T., Nines R.G., Shin H.C., Stoner G.D. (2006). Photochemoprevention of UVB-induced skin carcinogenesis in SKH-1 mice by brown algae polyphenols. Int. J. Cancer.

[60] Heo S.J., Ko S.C., Cha S.H., Kang D.H., Park H.S., Choi Y.U., Kim D., Jung W.K., Jeon Y.J. (2009). Effect of phlorotannins isolated from *Ecklonia cava* on melanogenesis and their protective effect against photo-oxidative stress induced by UV-B radiation. Toxicol. In Vitro.

[61] Cha S.H., Ko C.I., Kim D., Jeon Y.J. (2012). Protective effects of phlorotannins against ultraviolet B radiation in zebrafish (*Danio rerio*). Vet. Dermatol..

[62] Park E., Lee N.H., Joo H.G., Jee Y. (2008). Modulation of apoptosis of eckol against ionizing radiation in mice. Biochem. Biophys. Res. Commun..

[63] Moon C., Kim S.H., Kim J.C., Hyun J.W., Lee N.H., Park J.W., Shin T. (2008). Protective effect of phlorotannin components phloroglucinol and eckol on radiation-induced intestinal injury in mice. Phytother. Res..

[64] Park E., Ahn G.N., Lee N.H., Kim J.M., Yun J.S., Hyun J.W., Jeon Y.J., Wie M.B., Lee Y.J., Park J.W. (2008). Radioprotective properties of eckol against ionizing radiation in mice. FEBS Lett..

[65] Zhang R., Kang K.A., Piao M.J., Ko D.O., Wang Z.H., Lee I.K., Kim B.J., Jeong I.Y., Shin T., Park J.W. (2008). Eckol protects V79-4 lung fibroblast cells against γ-ray radiation-induced apoptosis via the scavenging of reactive oxygen species and inhibiting of the c-Jun NH2-terminal kinase pathway. Eur. J. Pharmacol..

[66] Ryu Y.B., Jeong H.J., Yoon S.Y., Park J.Y., Kim Y.M., Park S.J., Rho M.C., Kim S.J., Lee W.S. (2011). Influenza virus neuraminidase inhibitory activity of phlorotannins from the edible brown alga *Ecklonia cava*. J. Agric. Food Chem..

[67] Yang H.K., Jung M.H., Avunje S., Nikapitiya C., Kang S.Y., Ryu Y.B., Lee W.S., Jung S.J. (2018). Efficacy of algal *Ecklonia cava* extract against viral hemorrhagic septicemia virus (VHSV). Fish. Shellfish Immunol..

[68] Park J.Y., Kim J.H., Kwon J.M., Kwon H.J., Jeong H.J., Kim Y.M., Kim D., Lee W.S., Ryu Y.B. (2013). Dieckol, a SARS-CoV 3CL (pro) inhibitor, isolated from the edible brown algae *Ecklonia cava*. Bioorg. Med. Chem..

[69] Kim T.H., Lee T., Ku S.K., Bae J.S. (2012). Vascular barrier protective effects of eckol and its derivatives. Bioorg. Med. Chem. Lett..

[70] Sugiura Y., Usui M., Katsuzaki H., Imai K., Kakinuma M., Amano H., Miyata M. (2018). Orally administered phlorotannins from *Eisenia arborea* suppress chemical mediator release and cyclooxygenase-2 signaling to alleviate mouse ear swelling. Mar. Drugs.

[71] Eom S.H., Lee E.H., Park K., Kwon J.Y., Kim P.H., Jung W.K., Kim Y.M. (2017). Eckol from *Eisenia bicyclis* inhibits inflammation through the Akt/NF-κB signaling in *Propionibacterium acnes*-induced human keratinocyte HaCaT cells. J. Food Biochem..

[72] Shibata T., Nagayama K., Tanaka R., Yamaguchi K., Nakamura T. (2003). Inhibitory effects of brown algal phlorotannins on secretory phospholipase A_2_s, lipoxygenases and cyclooxygenases. J. Appl. Phycol..

[73] Li Y., Lee S.H., Le Q.T., Kim M.M., Kim S.K. (2008). Anti-allergic effects of phlorotannins on histamine release via binding inhibition between IgE and FcεRI. J. Agric. Food Chem..

[74] Shibata T., Fujimoto K., Nagayama K., Yamaguchi K., Nakamura T. (2002). Inhibitory activity of brown algal phlorotannins against hyaluronidase. Int. J. Food Sci. Technol..

[75] Hyun K.H., Yoon C.H., Kim R.K., Lim E.J., An S., Park M.J., Hyun J.W., Suh Y., Kim M.J., Lee S.J. (2011). Eckol suppresses maintenance of stemness and malignancies in glioma stem-like cells. Toxicol. Appl. Pharmacol..

[76] Henry M.M., Wilson M.N., Martin O.O., Ntevheleni T., Wilfred T.M. (2017). Phlorotannins and a sterol isolated from a brown alga *Ecklonia maxima*, and their cytotoxic activity against selected cancer cell lines HeLa, H157 and MCF7. Interdiscip. J. Chem..

[77] Kim T.H., Ku S.K., Bae J.S. (2012). Antithrombotic and profibrinolytic activities of eckol and dieckol. J. Cell. Biochem..

[78] Park E.Y., Kim E.H., Kim M.H., Seo Y.W., Lee J.I., Jun H.S. (2012). Polyphenol-rich fraction of brown alga *Ecklonia cava* collected from Gijang, Korea, reduces obesity and glucose levels in high-fat diet-induced obese mice. Evid. Based Complement. Alternat. Med..

[79] Eom S.H., Lee M.S., Lee E.W., Kim Y.M., Kim T.H. (2013). Pancreatic lipase inhibitory activity of phlorotannins isolated from *Eisenia bicyclis*. Phytother. Res..

[80] Yoon N.Y., Kim H.R., Chung H.Y., Choi J.S. (2008). Anti-hyperlipidemic effect of an edible brown algae, *Ecklonia stolonifera*, and its constituents on poloxamer 407-induced hyperlipidemic and cholesterol-fed rats. Arch. Pharm. Res..

[81] Moon H.E., Ahn B.R., Jung H.A., Choi J.S. (2012). Inhibitory activity of *Ecklonia stolonifera* and its isolated phlorotannins against Cu 2+-induced low-density lipoprotein oxidation. Fish. Sci..

[82] Kang H.S., Kim H.R., Byun D.S., Son B.W., Nam T.J., Choi J.S. (2004). Tyrosinase inhibitors isolated from the edible brown alga *Ecklonia stolonifera*. Arch. Pharm. Res..

[83] Agostinho P., Cunha R.A., Oliveira C. (2010). Neuroinflammation, oxidative stress and the pathogenesis of Alzheimer’s disease. Curr. Pharm. Des..

[84] Singh N., Ghosh K.K. (2019). Recent advances in the antioxidant therapies for Alzheimer’s disease: Emphasis on natural antioxidants. Pathology, Prevention and Therapeutics of Neurodegenerative Disease.

[85] Ceriello A., Esposito K., Piconi L., Ihnat M.A., Thorpe J.E., Testa R., Boemi M., Giugliano D. (2008). Oscillating glucose is more deleterious to endothelial function and oxidative stress than mean glucose in normal and type 2 diabetic patients. Diabetes.

[86] Perše M. (2013). Oxidative stress in the pathogenesis of colorectal cancer: Cause or consequence?. BioMed Res. Int..

[87] Trouba K.J., Hamadeh H.K., Amin R.P., Germolec D.R. (2002). Oxidative stress and its role in skin disease. Antioxid. Redox Signal..

[88] McCall A.L. (2012). Insulin therapy and hypoglycemia. Endocrinol. Metab. Clin..

[89] Okada Y., Ishimaru A., Suzuki R., Okuyama T. (2004). A new phloroglucinol derivative from the brown alga *Eisenia bicyclis*: Potential for the effective treatment of diabetic complications. J. Nat. Prod..

[90] Moon H.E., Islam M.N., Ahn B.R., Chowdhury S.S., Sohn H.S., Jung H.A., Choi J.S. (2011). Protein tyrosine phosphatase 1B and α-glucosidase inhibitory phlorotannins from edible brown algae, *Ecklonia stolonifera* and *Eisenia bicyclis*. Biosci. Biotechnol. Biochem..

[91] Agarwal S., Manchanda P., Vogelbaum M.A., Ohlfest J.R., Elmquist W.F. (2013). Function of the blood-brain barrier and restriction of drug delivery to invasive glioma cells: Findings in an orthotopic rat xenograft model of glioma. Drug Metab. Dispos..

[92] Osseni R., Debbasch C., Christen M.O., Rat P., Warnet J.M. (1999). Tacrine-induced reactive oxygen species in a human liver cell line: The role of anethole dithiolethione as a scavenger. Toxicol. In Vitro.

[93] Heemels M.T. (2016). Neurodegenerative diseases. Nature.

[94] Schliebs R., Arendt T. (2011). The cholinergic system in aging and neuronal degeneration. Behav. Brain Res..

[95] Goldman J.G., Postuma R. (2014). Premotor and non-motor features of Parkinson’s disease. Curr. Opin. Neurol..

[96] Ingelsson M. (2016). Alpha-synuclein oligomers—neurotoxic molecules in Parkinson’s disease and other lewy body disorders. Front. Neurosci..

[97] Meletis C.D., Zabriskie N. (2008). Natural approaches for optimal sleep. Altern. Complement. Ther..

[98] Hauser A.S., Attwood M.M., Rask-Andersen M., Schiöth H.B., Gloriam D.E. (2017). Trends in GPCR drug discovery: New agents, targets and indications. Nat. Rev. Drug Discov..

[99] Wang S., Wacker D., Levit A., Che T., Betz R.M., McCorvy J.D., Venkatakrishnan A., Huang X.P., Dror R.O., Shoichet B.K. (2017). D4 dopamine receptor high-resolution structures enable the discovery of selective agonists. Science.

[100] Chien E.Y., Liu W., Zhao Q., Katritch V., Han G.W., Hanson M.A., Shi L., Newman A.H., Javitch J.A., Cherezov V. (2010). Structure of the human dopamine D3 receptor in complex with a D2/D3 selective antagonist. Science.

[101] Mashiba H., Matsunaga K., Gojobori M. (1979). Effect of immunochemotherapy with OK-432 and yeast cell wall on the activities of peritoneal macrophages of mice. Jpn. J. Cancer Res..

[102] Bogo V., Jacobs A., Weiss J. (1985). Behavioral toxicity and efficacy of WR-2721 as a radioprotectant. Radiat. Res..

[103] Jagetia G.C. (2007). Radioprotective potential of plants and herbs against the effects of ionizing radiation. J. Clin. Biochem. Nutr..

[104] Rabe J.H., Mamelak A.J., McElgunn P.J., Morison W.L., Sauder D.N. (2006). Photoaging: Mechanisms and repair. J. Am. Acad. Dermatol..

[105] Lavker R. (1995). Cutaneous aging: Chronologic versus photoaging. Photodamage.

[106] Gilchrest B.A. (1989). Skin aging and photoaging: An overview. J. Am. Acad. Dermatol..

[107] Quan T., Qin Z., Xia W., Shao Y., Voorhees J.J., Fisher G.J. (2009). Matrix-degrading metalloproteinases in photoaging. J. Investig. Dermatol. Symp. Proc..

[108] Nelson A.R., Fingleton B., Rothenberg M.L., Matrisian L.M. (2000). Matrix metalloproteinases: Biologic activity and clinical implications. J. Clin. Oncol..

[109] Sternlicht M.D., Werb Z. (2001). How matrix metalloproteinases regulate cell behavior. Annu. Rev. Cell Dev. Biol..

[110] Birkedal-Hansen H., Moore W., Bodden M., Windsor L., Birkedal-Hansen B., DeCarlo A., Engler J. (1993). Matrix metalloproteinases: A review. Crit. Rev. Oral Biol. Med..

[111] Egeblad M., Werb Z. (2002). New functions for the matrix metalloproteinases in cancer progression. Nat. Rev. Cancer.

[112] Kerkelä E., Saarialho-Kere U. (2003). Matrix metalloproteinases in tumor progression: Focus on basal and squamous cell skin cancer. Exp. Dermatol..

[113] Fisher G.J., Datta S., Wang Z., Li X.Y., Quan T., Chung J.H., Kang S., Voorhees J.J. (2000). c-Jun–dependent inhibition of cutaneous procollagen transcription following ultraviolet irradiation is reversed by all-trans retinoic acid. J. Clin. Investig..

[114] Vincenti M.P., White L.A., Schroen D.J., Benbow U., Brinckerhoff C.E. (1996). Regulating expression of the gene for matrix metalloproteinase-1 (collagenase): Mechanisms that control enzyme activity, transcription, and mRNA stability. Crit. Rev. Eukaryot. Gene Expr..

[115] Scharffetter–Kochanek K., Brenneisen P., Wenk J., Herrmann G., Ma W., Kuhr L., Meewes C., Wlaschek M. (2000). Photoaging of the skin from phenotype to mechanisms. Exp. Gerontol..

[116] Scharffetter-Kochanek K., Wlaschek M., Brenneisen P., Schauen M., Blaudschun R., Wenk J. (1997). UV-induced reactive oxygen species in photocarcinogenesis and photoaging. Biol. Chem..

[117] Kvam E., Tyrrell R.M. (1997). Induction of oxidative DNA base damage in human skin cells by UV and near visible radiation. Carcinogenesis.

[118] Atkinson A., Robertson J. (1979). Captopril in the treatment of clinical hypertension and cardiac failure. Lancet.

[119] Wijesinghe W., Ko S.C., Jeon Y.J. (2011). Effect of phlorotannins isolated from *Ecklonia cava* on angiotensin I-converting enzyme (ACE) inhibitory activity. Nutr. Res. Pract..

[120] Fukuyama Y., Kodama M., Miura I., Kinzyo Z., Kido M., Mori H., Nakayama Y., Takahashi M. (1989). Structure of an anti-plasmin inhibitor, eckol, isolated from the brown alga *Ecklonia kurome* Okamura and inhibitory activities of its derivatives on plasma plasmin inhibitors. Chem. Pharm. Bull..

[121] Wise R., Hart T., Cars O., Streulens M., Helmuth R., Huovinen P., Sprenger M. (1998). Antimicrobial resistance. Is a major threat to public health. Br. Med. J..

[122] Pérez M., Falqué E., Domínguez H. (2016). Antimicrobial action of compounds from marine seaweed. Mar. Drugs.

[123] Eom S.H., Lee D.S., Kang Y.M., Son K.T., Jeon Y.J., Kim Y.M. (2013). Application of yeast *Candida utilis* to ferment *Eisenia bicyclis* for enhanced antibacterial effect. Appl. Biochem. Biotechnol..

[124] Lee J.H., Eom S.H., Lee E.H., Jung Y.J., Kim H.J., Jo M.R., Son K.T., Lee H.J., Kim J.H., Lee M.S. (2014). In vitro antibacterial and synergistic effect of phlorotannins isolated from edible brown seaweed *Eisenia bicyclis* against acne-related bacteria. Algae.

[125] Ahn M.J., Yoon K.D., Min S.Y., Lee J.S., Kim J.H., Kim T.G., Kim S.H., Kim N.G., Huh H., Kim J. (2004). Inhibition of HIV-1 reverse transcriptase and protease by phlorotannins from the brown alga *Ecklonia cava*. Biol. Pharm. Bull..

[126] Obici S., Rossetti L. (2003). Minireview: Nutrient sensing and the regulation of insulin action and energy balance. Endocrinology.

[127] Gurevich-Panigrahi T., Panigrahi S., Wiechec E., Los M. (2009). Obesity: Pathophysiology and clinical management. Curr. Med. Chem..

[128] Vazzana N., Santilli F., Sestili S., Cuccurullo C., Davi G. (2011). Determinants of increased cardiovascular disease in obesity and metabolic syndrome. Curr. Med. Chem..

[129] Youssef W.I., McCullough A.J. (2002). Steatohepatitis in obese individuals. Best Pract. Res. Clin. Gastroenterol..

[130] Lowe M.E. (1994). Pancreatic triglyceride lipase and colipase: Insights into dietary fat digestion. Gastroenterology.

[131] Croce C.M. (2008). Oncogenes and cancer. N. Engl. J. Med..

[132] Ferlay J., Soerjomataram I., Dikshit R., Eser S., Mathers C., Rebelo M., Parkin D.M., Forman D., Bray F. (2015). Cancer incidence and mortality worldwide: Sources, methods and major patterns in GLOBOCAN 2012. Int. J. Cancer..

[133] Lagunoff D., Martin T., Read G. (1983). Agents that release histamine from mast cells. Annu. Rev. Pharmacol. Toxicol..

[134] Church M.K., Levi-Schaffer F. (1997). The human mast cell. J. Allergy Clin. Immunol..

[135] Xie Q.W., Whisnant R., Nathan C. (1993). Promoter of the mouse gene encoding calcium-independent nitric oxide synthase confers inducibility by interferon gamma and bacterial lipopolysaccharide. J. Exp. Med..

[136] He P. (2010). Leucocyte/endothelium interactions and microvessel permeability: Coupled or uncoupled?. Cardiovasc. Res..

[137] Komarova Y.A., Mehta D., Malik A.B. (2007). Dual regulation of endothelial junctional permeability. Sci. STKE.

[138] Majno G., Palade G. (1961). Studies on inflammation: I. The effect of histamine and serotonin on vascular permeability: An electron microscopic study. J. Biophys. Biochem. Cytol..

[139] Ware L.B., Matthay M.A. (2000). The acute respiratory distress syndrome. N. Engl. J. Med..

[140] Dhillon S.S., Mahadevan K., Bandi V., Zheng Z., Smith C.W., Rumbaut R.E. (2005). Neutrophils, nitric oxide, and microvascular permeability in severe sepsis. Chest.

[141] Cummings B.S., McHowat J., Schnellmann R.G. (2000). Phospholipase A2s in cell injury and death. J. Pharmacol. Exp. Ther..

[142] Bomalaski J.S., Clark M.A. (1993). Phospholipase A2 and arthritis. Arthritis Rheum..

[143] Liu P.F., Nakatsuji T., Zhu W., Gallo R.L., Huang C.M. (2011). Passive immunoprotection targeting a secreted CAMP factor of *Propionibacterium acnes* as a novel immunotherapeutic for acne vulgaris. Vaccine.

[144] Choi J.Y., Piao M.S., Lee J.B., Oh J.S., Kim I.G., Lee S.C. (2008). Propionibacterium acnes stimulates pro-matrix metalloproteinase-2 expression through tumor necrosis factor-α in human dermal fibroblasts. J. Investig. Dermatol..

[145] Jalian H.R., Liu P.T., Kanchanapoomi M., Phan J.N., Legaspi A.J., Kim J. (2008). All-trans retinoic acid shifts *Propionibacterium acnes*-induced matrix degradation expression profile toward matrix preservation in human monocytes. J. Investig. Dermatol..

[146] Stevens L.H., Davelaar E., Kolb R.M., Pennings E.J., Smit N.P. (1998). Tyrosine and cysteine are substrates for blackspot synthesis in potato. Phytochemistry.

[147] Ando H., Kondoh H., Ichihashi M., Hearing V.J. (2007). Approaches to identify inhibitors of melanin biosynthesis via the quality control of tyrosinase. J. Investig. Dermatol..

[148] Lee S.H., Kang S.M., Sok C.H., Hong J.T., Oh J.Y., Jeon Y.J. (2015). Cellular activities and docking studies of eckol isolated from *Ecklonia cava* (Laminariales, Phaeophyceae) as potential tyrosinase inhibitor. Algae.

[149] Kang J.I., Kim S.C., Kim M.K., Boo H.J., Jeon Y.J., Koh Y.S., Yoo E.S., Kang S.M., Kang H.K. (2012). Effect of dieckol, a component of *Ecklonia cava*, on the promotion of hair growth. Int. J. Mol. Sci..

[150] Chang M.Y., Han S.Y., Shin H.C., Byun J.Y., Rah Y.C., Park M.K. (2016). Protective effect of a purified polyphenolic extract from *Ecklonia cava* against noise-induced hearing loss: Prevention of temporary threshold shift. Int. J. Pediatr. Otorhinolaryngol..

[151] Djouad F., Guerit D., Marie M., Toupet K., Jorgensen C., Noel D. (2012). Mesenchymal stem cells: New insights into bone regenerative applications. J. Biomater. Tissue Eng..

[152] Gimble J.M., Nuttall M.E. (2012). The relationship between adipose tissue and bone metabolism. Clin. Biochem..

[153] Yamaguchi M. (2013). Bone marrow mesenchymal stem cell differentiation: Involvement in osteoporosis with obesity and diabetes. J. Bone Marrow Res..

[154] Karadeniz F., Ahn B.N., Kim J.A., Seo Y., Jang M.S., Nam K.H., Kim M., Lee S.H., Kong C.S. (2015). Phlorotannins suppress adipogenesis in pre-adipocytes while enhancing osteoblastogenesis in pre-osteoblasts. Arch. Pharm. Res..

